# Rejuvenating effects of young extracellular vesicles in aged rats and in cellular models of human senescence

**DOI:** 10.1038/s41598-023-39370-5

**Published:** 2023-07-28

**Authors:** Lilian Grigorian Shamagian, Russell G. Rogers, Kristin Luther, David Angert, Antonio Echavez, Weixin Liu, Ryan Middleton, Travis Antes, Jackelyn Valle, Mario Fourier, Liz Sanchez, Eva Jaghatspanyan, Javier Mariscal, Rui Zhang, Eduardo Marbán

**Affiliations:** 1 Cedars-Sinai Medical Center, Smidt Heart Institute, Los Angeles, CA USA; 2 Servicio de Cardiología, Hospital Gregorio Marañón, Instituto de Investigación Sanitaria Gregorio Marañón, Universidad Complutense, c/O’Donnell 48-50 (planta -1), 28009 Madrid, Spain; 3 CIBERCV, ISCIII, Madrid, Spain; 4 Samuel Oschin Comprehensive Cancer Institute, Los Angeles, CA USA

**Keywords:** Ageing, Cardiac regeneration

## Abstract

Rejuvenation of an old organism was achieved in heterochronic parabiosis experiments, implicating different soluble factors in this effect. Extracellular vesicles (EVs) are the secretory effectors of many cells, including cardiosphere-derived cells (CDCs) with demonstrated anti-senescent effect. 1. To determine the role of EVs (versus other blood fractions) on the rejuvenating effect of the young blood. 2. To evaluate the anti-aging properties of therapeutically administered EVs secreted by young-CDCs in an old organism. Neonatal blood fractioned in 4 components (whole blood, serum, EV-depleted serum and purified EVs) was used to treat old human cardiac stromal cells (CSPCs). CDCs were generated from neonatal rat hearts and the secreted CDC-EVs were purified. CDC-EVs were then tested in naturally-aged rats, using monthly injections over 4-months period. For validation in human samples, pediatric CDC-EVs were tested in aged human CSPCs and progeric fibroblasts. While the purified EVs reproduced the rejuvenating effects of the whole blood, CSPCs treated with EV-depleted serum exhibited the highest degree of senescence. Treatment with young CDC-EVs induce structural and functional improvements in the heart, lungs, skeletal muscle, and kidneys of old rats, while favorably modulating glucose metabolism and anti-senescence pathways. Lifespan was prolonged. EVs secreted by young CDCs exert broad-ranging anti-aging effects in aged rodents and in cellular models of human senescence. Our work not only identifies CDC-EVs as possible therapeutic candidates for a wide range of age-related pathologies, but also raises the question of whether EVs function as endogenous modulators of senescence.

## Introduction

By 2050, nearly 1.5 billion people will be over age 65^1,2^, outnumbering children. As a consequence, aging-related diseases, including cardiovascular disease, diabetes, and cancer, will increase dramatically in the coming decades. Today we treat these chronic conditions by addressing individual disease-specific pathways, rather than by targeting cellular senescence as a potentially treatable pathogenic mechanism. Cellular senescence is the phenomenon believed to underlie progressive aging-related tissue and organ dysfunction^3,4^.

Cardiosphere-derived cells (CDCs) are cardiac progenitor cells with broad-ranging bioactivity in preclinical and clinical studies^5–9^. Recently, we found that transplantation of young CDCs exerts anti-aging effects in old rodents, improving heart function^10^. Although we specifically targeted the heart in that study, multiple systemic benefits were evident, hinting that soluble factors might play a prominent role. In vitro experiments revealed that extracellular vesicles (EVs) from CDCs (CDC-EVs) mimicked the anti-senescent effects of CDCs, at least partially through activation of the telomerase-telomere axis. Together with the anti-tumorigenic effects of CDC-EVs in old rats with spontaneous leukemia^11^, it seems reasonable to hypothesize that anti-senescent properties of CDC-EVs may underlie the benefits. If so, EVs might be logical therapeutic candidates for a variety of aging-related diseases.

Here, we address the following questions: Do CDC-EVs exert anti-aging properties in vivo? Are favorable changes durable, and do CDC-EVs affect longevity? Finally, do EVs (versus other blood fractions) mediate anti-senescent effects of young blood observed in heterochronic parabiosis^12^?

## Methods

For detailed methods, please see the supplementary file.

### In vivo experiments

Twenty-two-month-old Fisher 344 rats (male and female) were obtained from the National Institute of Aging. All methods were carried out in accordance with relevant guidelines, approved by the Institutional Animal Care and Use Committee (IACUC) of the Cedars-Sinai Medical Center as per the National Institutes of Health (NIH Publication No. 86–23, revised 1996), and are reported in accordance with ARRIVE guidelines. Old Fisher 344 rats were selected for this study since they mimic different human aging-related conditions such as cardiac fibrosis and diastolic dysfunction^10,13^. After initial evaluation with echocardiography and exercise testing, a total of 27 animals were divided randomly into two groups^1^: 13 rats treated with extracellular vesicles secreted by neonatal cardiosphere-derived cells (CDC-EVs)^2^, 14 rats receiving phosphate buffered saline (PBS, i.e. vehicle control). CDCs were grown from 30 freshly-explanted F344 neonatal rat hearts as described (suppl. ref.1). CDC-EVs were harvested from passage 2 CDCs, from serum-free medium conditioned for 15 days. An initial dose of 7.5 μg EV protein/g animal weight (based on our previous results^11^), equivalent to 1.3 × 10^8^ particles/g animal weight followed by lower monthly doses (1.5 μg/g, equivalent to 2.6 × 10^7^ particles/g) of CDC-EVs (resuspended in 300 µL PBS) or 300 µL PBS alone, injected percutaneously into the left ventricular (LV) cavity for systemic delivery. Cardiac echocardiography and exercise test were performed monthly and hemodynamic measurements were performed at endpoint. Blood samples were collected at baseline and endpoint. When appropriate (see supplementary file) animals received anesthesia with inhaled Isoflurane 4% for induction followed by 2%. After 16-weeks of follow-up animals were sacrificed, under general anesthesia hearts were arrested in diastole (intra-ventricular injection of KCl) and excised. Hearts and other organs were collected. In a separate group of 11 male and female 22-month-old Fisher 344 rats, glucose metabolism was evaluated with the glucose tolerance test (GTT).

### In vitro studies

For human cells, the protocol was deemed exempt by the Institutional Review Board of the Cedars-Sinai Medical Center as it only involved the use of anonymized pathological specimens for tissue harvesting and CDC isolation. Old human cardiac stromal cells and human progeric fibroblasts were targeted with CDC-EVs isolated from human pediatric donors. Simulating heterochronic parabiosis in vitro, old human cardiac stromal cells were treated with different fractions of neonatal rat blood. Four different blood fractions were used in the consecutive experiments: total blood with cells, serum, EVs-depleted serum and purified EVs.

### Statistical analysis

Pooled results are presented as mean ± SD (± SEM in figures) or percentages, for continuous and categorical variables, respectively. Significance of differences was assessed by Student t test or with 1-way ANOVA in case of multiple groups if the distribution of the variable was normal; otherwise, the Mann-Whitney or Kruskal-Wallis tests were used. Paired t-test was used to determine significance between baseline and endpoint in the same group of animals. All probability values reported are 2-sided, with *p* < 0.05 considered significant. IBM SPSS Statistics 20 was used for all analyses. For in vitro studies the lowest number of replicates per experiment was three.

## Results

**Figure 1:**
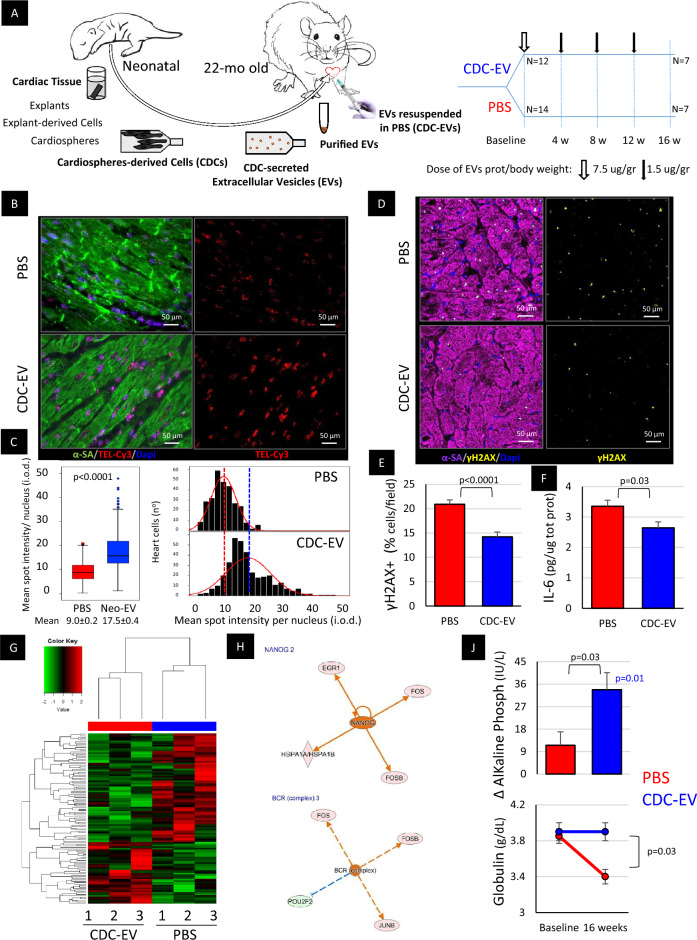
Signs of heart rejuvenation in old rats treated with extracellular vesicles secreted by neonatal cardiosphere-derived cells (CDC-EVs). (**A**) A schematic of the main study protocol. Heart explant-derived cells from thirty F344 rat pups were pooled together to generate CDCs. CDC-EVs were purified from serum-free medium conditioned for 15 days by CDCs and resuspended in phosphate-buffered saline (PBS). 22-month-old F344 rats after an initial evaluation were randomized to receive percutaneous intra-left ventricular injection of CDC-EVs resuspended in PBS or PBS alone (300 μL). A higher “loading” dose of CDC-EVs was followed by lower consecutive monthly doses. (**B**) Telomere length in heart cells. Representative detail of confocal maximum projection images of telomere Q-FISH (TEL-Cy3) and alpha-sarcomeric actinin (α-SA) immunofluorescence in old animals treated with PBS (n = 6) and CDC-EVs (n = 5). (**C**) Mean telomere length and telomere length distribution in all cells analyzed. (**D**) Representative image of confocal micrographs of heart sections stained for phosphorylated histone H2A (γH2AX), and α-SA in old animals treated with PBS (n = 7) and CDC-EVs (n = 7). (**E**) The proportion of γH2AX+ cells is lower in CDC-EV versus PBS treated rats. (**F**) Normalized expression of IL-6 in heart tissue protein lysates in PBS (n = 7) and CDC-EV (n = 7) treated rats. (**G**) Next generation RNA sequencing of heart tissue in randomly selected animals from PBS and CDC-EV groups (n = 3 in each). The heat map shows transcriptomic differences between the groups. (**H**) Ingenuity pathway analysis identified pluripotency transcription factor NANOG and B-cell receptor (BCR) complex (composed by CD79 and immunoglobulin) as upstream regulators of gene expression differences observed in CDC-EV-injected rats versus PBS group. (**J**) Serum levels of alkaline phosphatase (AP) and globulin. All significant *p*-values are shown. The blue value is related with the changes in a parameter between baseline and endpoint within the CDC-EV group, estimated with paired Student’s t-test. Black values are related with the differences between the groups at the same time point, estimated with two tailed Student’s t-test. Data presented are means ± s.e.m.

CDC-EVs were isolated from neonatal rat CDCs as described in Methods and Fig. 1A. CDC-EVs resuspended in PBS, or PBS alone, were injected percutaneously under ultrasound guidance into the LV lumen of 22-month-old Fisher 344 rats (“old rats”) on a monthly basis, to achieve systemic arterial delivery. Animals were followed for 16 weeks and survivors were euthanized for further studies.

### CDC-EV effects in aged hearts: from tissue rejuvenation to functional improvement

After 4 monthly systemic injections, the hearts of CDC-EV-treated rats, but not control rats, showed signs of rejuvenation: telomere length (~two-fold increase, *p *< 0.001; Fig. 1B,C), phosphorylated histone H2A (γH2AX; 32% decrease, *p* < 0.001; Fig. 1D,E, Suppl Figure 2A), and interleukin 6 (IL-6; 21% decrease, *p* = 0.03; Fig. 1F), a component of the senescence-associated secretory phenotype^14^, were all favorably modulated. Telomere length was ~2 times longer in CDC-EV treated versus control rats, as in our previous study using CDCs^10^. Ingenuity pathway analysis of the transcriptomes from CDC-EV and control rat hearts (Fig. 1G) identified changes in 3 major upstream regulators (Fig. 1H and Suppl Figure 2B): NANOG, a pluripotency marker and cell reprogramming enhancer (*z* = 2; *p* = 0.001)^15,16^; immune B-cell receptor complex (BCR, z = 2; *p* < 0.001); and extracellular signal-regulated protein kinases 1 and 2 (ERK1/2, z = 2.6; *p* < 0.001). In the serum of CDC-EV treated rats, we observed an increase in alkaline phosphatase (34 IU/L, *p* = 0.01) and preservation of globulin levels compared with controls (Fig. 1J). These may be associated with an increase in circulating stem/progenitor cells^17^ and preservation of immunity^18^ in CDC-EV-treated rats, respectively, but we have not tested these conjectures.

**Figure 2:**
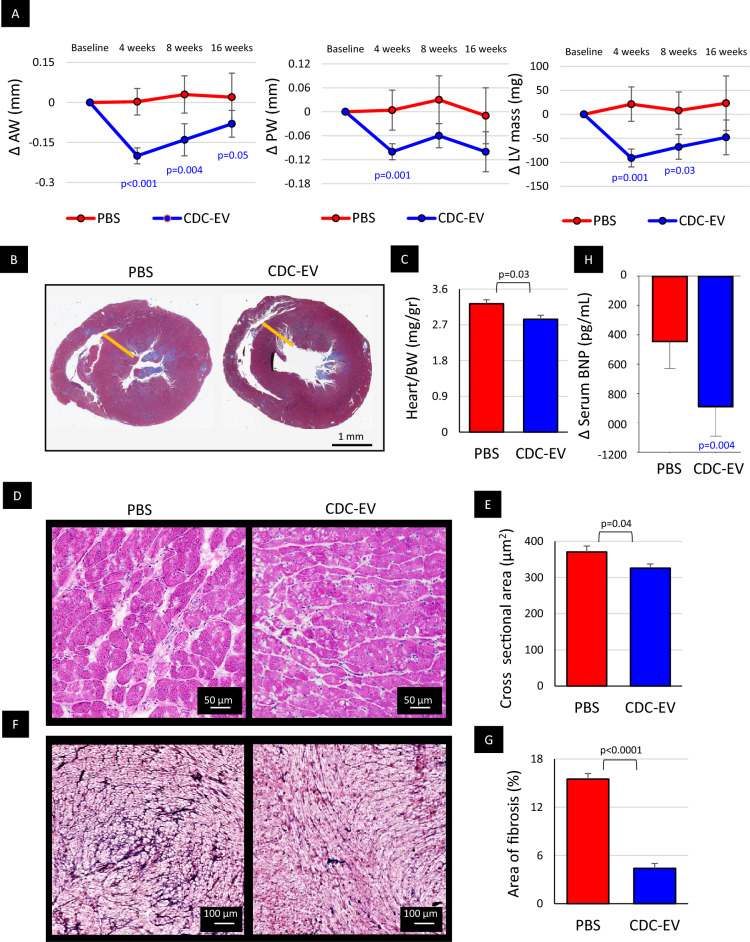
Heart structure is improved and BNP levels are decreased in old rats treated with extracellular vesicles secreted by neonatal cardiosphere-derived cell (CDC-EVs). (**A**) CDC-EV-injected rats (n = 12) had decreased thickness of the anterior and posterior left ventricular (LV) walls and reduced LV mass. Control PBS rats (n = 14) showed an opposite trend. (**B**) Representative mid-heart sections from a rat in each group. (**C**) Heart/body weight ratio (BW) was lower in CDC-EV (n = 6) versus PBS (n = 7) treated rats. (**D**) Histological sections of myocardium from a rat in each group stained with hematoxylin and eosin. (**E**) Pooled data for cardiomyocyte cross sectional area in CDC-EV-injected (n = 6) versus PBS-injected (n = 6) rats. (**F**) Representative heart sections stained with Masson’s trichrome. (**G**) The CDC-EV-group (n = 7) exhibited a decrease of fibrosis versus control PBS (n = 6). (**H**) The decrease in serum levels of BNP was significant after 16 weeks of treatment in CDC-EV group (n = 7) while in the control PBS rats (n = 7), the change was not significant. AW: left ventricular anterior wall; PW: left ventricular posterior wall; LV: left ventricle. All significant p-values are represented. Blue values (CDC-EV group) represent the significance of the difference between baseline and the specific time-point within the group estimated with paired Student’s t-test. Black values represent the significance between the groups at the same time point estimated with two tailed Student’s t-test. Data presented are means ± s.e.m.

Echocardiography showed regression of LV hypertrophy in CDC-EV treated rats but not controls (14.5% decrease in LV mass at 1-month follow-up, *p* = 0.001; Fig. 2A, Suppl. Figure 3), as verified by post-mortem macroscopic evaluation (12.1% decrease of heart/body weight ratio; *p* = 0.03; Fig. 2B,C) and histology (12.0% decrease of cardiomyocytes cross sectional area; *p* = 0.04; Fig. 2D,E). Likewise, myocardial fibrosis was attenuated in CDC-EV-injected rats relative to controls (by 70%; *p *< 0.001; Fig. 2F,G). Significant reductions in serum levels of brain natriuretic peptide (BNP), seen only in CDC-EV-injected rats, attest to the systemic hemodynamic impact of the structural findings (~900 pg/mL; *p *< 0.01; Fig. 2H).

**Figure 3:**
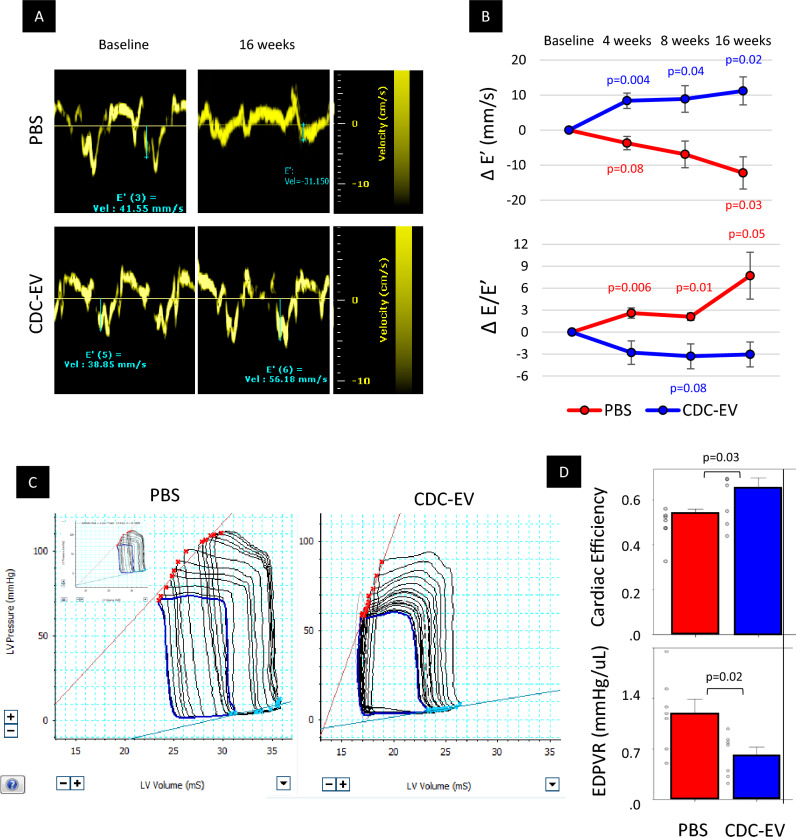
Echocardiographic and hemodynamic evaluation of heart functional parameters. (**A**) Representative images of mitral annulus tissue Doppler in a rat from the phosphate-buffered saline (PBS-control) and a rat from the CDC-EV treated groups. (**B**) The CDC-EV group showed a trend towards decreased LV stiffness compared with the control group, as measured by E′ and E/E′ ratio. (**C**) Representative images of left ventricular (LV) pressure-volume loops (PVL) in a rat from PBS-control (n = 7) and CDC-EV-treated (n = 7) groups. (**D**) Cardiac efficiency was higher and LV end-diastolic pressure-volume relationship (EDPVR) slopes were lower in CDC-EV-treated group after 16 weeks versus control PBS. All significant p-values are shown. Colored values (blue in CDC-EV and red in PBS groups, respectively) represent the significance of the difference between baseline and the specific time-point within the group estimated with paired Student’s t-test. Black values represent the significance between the groups at the same time-point estimated with two tailed Student’s t-test. Data presented are means ± s.e.m.

Both serial echocardiography and endpoint invasive hemodynamics revealed reduced LV stiffness (i.e., improvement of diastolic function) in rats treated with CDC-EVs. Echocardiography showed progressive changes of E′ and E/E′ ratio, with improvements in the CDC-EV group but deterioration in controls (E’-wave 11.2 ± 1.4 mm/s increase; *p* = 0.02 in CDC-EV group and 10.9 ± 12.8 mm/s decrease; *p *= 0.06 in PBS group at study end-point; Fig. 3A, B; Suppl. Figure 3). Elastance catheter analysis showed decreases in the slopes of the end-diastolic pressure–volume relationship in CDC-EV rats, but not controls (EDPVR; 50% decrease, *p* = 0.02; Fig. 3C,D-lower panel). Moreover, cardiac efficiency, a parameter reflective of myocardial metabolism^19^, was improved in CDC-EV versus control rats (28%, *p* = 0.03; Fig. 3C,D-upper panel).

### Metabolic effects of CDC-EVs in old rats

Rats injected with CDC-EVs lost weight over the 16 weeks of follow up (Suppl. Figure 4A), in association with decreased visceral abdominal fat on necropsy (Suppl. Figure 4B). Blood glucose levels tended to fall in CDC-EV rats, while the opposite tendency was seen in controls (Suppl. Figure 4C). Analysis of several proteins implicated in insulin sensitivity and oxidative metabolism in *biceps femoris* lysates revealed up-regulation in CDC-EV-rats versus controls (1.7-fold up-regulation of citrate synthase activity, *p* < 0.001; Suppl. Figure 4D, E, Suppl. Figure 8), consistent with higher mitochondrial biogenesis^20^. To assay the effects of CDC-EVs on glucose metabolism, we performed glucose tolerance tests at baseline and 4 weeks later (end-point) in a separate group of 22-month-old rats. The end-point test was done 48 h after CDC-EV or PBS infusion (Suppl. Figure 4F). In PBS-infused controls, the basal and follow-up responses of blood glucose were comparable, but glucose levels were significantly lower just 2 days after CDC-EV infusion (Suppl. Figure 4G). The reductions in visceral fat and the improved glucose tolerance support the idea that CDC-EVs may decrease age-related insulin resistance. In support of this hypothesis, CDC-EV rats displayed lower levels of fasting serum insulin and greater hyperglycemia-induced insulin secretion than controls (Suppl. Figure 4H).

### Other systemic effects of CDC-EVs in aged rats

**Figure 4:**
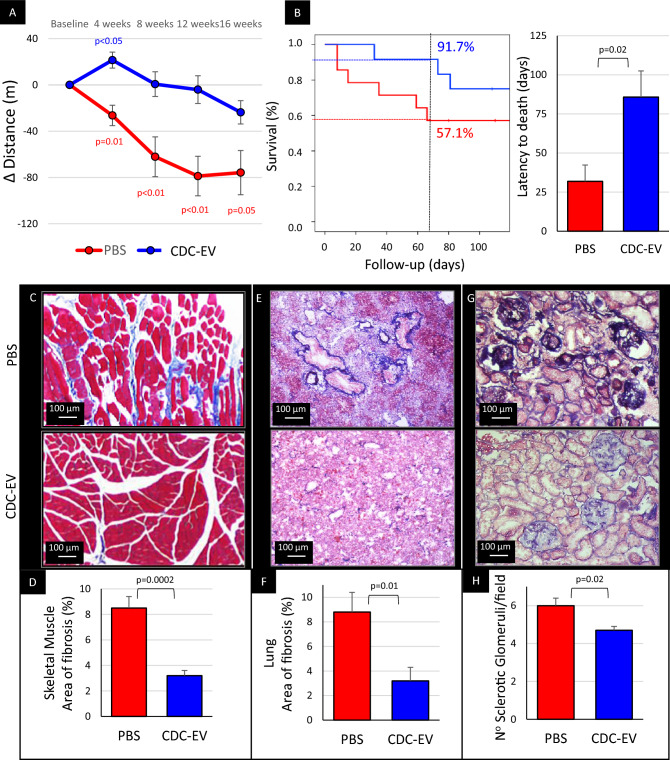
Extracellular vesicles secreted by neonatal cardiosphere-derived cells (CDC-EVs) induce broad systemic effects and prolong survival of old rats. (**A**) Changes in maximal exercise capacity over follow-up period show a steady decrease in the control group, but an initial improvement and subsequent preservation in CDC-EV-treated animals. (**B**) Kaplan-Meier survival curves show that after 65 days, 91.7% of rats survived in CDC-EV-group compared with 57.1% in PBS group; latency to death was nearly 3 times longer in vesicle-treated rats compared with placebo. (**C**) Representative lower limb skeletal muscle sections stained with Masson’s trichrome. (**D**) Pooled data shows that CDC-EV-group (n = 7) has less fibrosis than control PBS (n = 7). (**E**) Representative lung sections stained with Masson’s trichrome. (**F**) Pooled data shows that CDC-EV-group (n = 7) has less fibrosis than control PBS (n = 7). (**G**) Representative kidney sections stained with Masson’s trichrome. (**H**) Pooled data shows that CDC-EV-injected rats (n = 5) have lower number of sclerotic glomeruli/field versus PBS group (n = 5). All significant *p*-values are shown. Colored values (blue in CDC-EV and red in PBS groups, respectively) represent the significance of the difference between baseline and the specific time-point within the group estimated with paired Student’s t-test. Black values represent the significance between the groups at the same time-point estimated with two tailed Student’s t-test. Data presented are means ± s.e.m.

Exercise capacity is increased in aged rats after infusion of neonatal CDCs^10^. Likewise, CDC-EVs increased treadmill exercise capacity by ~16% in the month after the first dose (133 ± 51 m to 154 ± 61 m; *p* < 0.05) and remained higher than in the PBS group (which deteriorated progressively) throughout follow-up (Fig. 4A). Moreover, survival was improved in CDC-EV-treated rats versus PBS controls, with the latency to death increased by 54 days (Fig. 4B), which extrapolates to ~3.2 human years^21^. Spontaneous leukemia is a frequent cause of death in aged F344 rats^22^. Indeed, leukemia-related death occurred in 50% of PBS-injected rats versus 28% in the CDC-EV-injected animals, consistent with previous findings^11^.

Aging causes progressive replacement of healthy parenchyma by fibrotic tissue in a variety of organs, contributing to loss of function^23^. The heart, skeletal muscle, and lungs exhibited extensive fibrosis in the control rats (~8%) but decreased strikingly (to ~30% of control levels) in CDC-EV animals (Fig. 4C–G). Similarly, the kidneys of aged control rats had appreciable glomerulosclerosis, which is a common cause of renal dysfunction in old F344 rats^24^. The number of sclerotic glomeruli was lower in CDC-EV vs PBS-injected rats (Fig. 4G,H), rationalizing the improvements in circulating biomarkers of renal function in the CDC-EV group (Suppl. Figure 5).

### Anti-senescent effects of CDC-EVs are reproduced in aged human heart cells and progeric fibroblasts

To exclude rodent-specific effects, we tested rejuvenating effects of CDC-EVs from young human (donors < 2 years of age) on aged human cells. In vitro assays used cardiac stromal/progenitor cells (CSPC) obtained from > 55-year-old human donors and dermal fibroblasts from progeric patients. Aged human cells were treated with CDC-EVs resuspended in serum-free conditioned medium or serum-free medium alone (Methods).

Exposure of aged CSPC with young CDC-EVs triggered favorable modulation of DNA repair genes (Suppl. Figure 6A), down-regulation of protein effectors of the chronic senescence pathway^4,25^ and an increase in anti-oxidative proteins^26,27^ (Suppl. Figure 6B). Transcriptional and proteomic changes observed in CDC-EV primed CSPC versus control cells were associated with increased proliferation, decreased apoptosis (Suppl. Figure 6C, D, respectively), and increased self-assembly potential (Suppl. Figure 6E). The benefits were not limited to cells of cardiovascular origin: CDC-EV-primed progeric fibroblasts manifested rejuvenation in terms of increased proliferation, decreased number of senescent cells (Suppl. Figure 6F, G, respectively), and favorable changes in the transcriptome (Suppl. Figure 7).

### EVs are the primary bloodborne rejuvenating messengers

**Figure 5:**
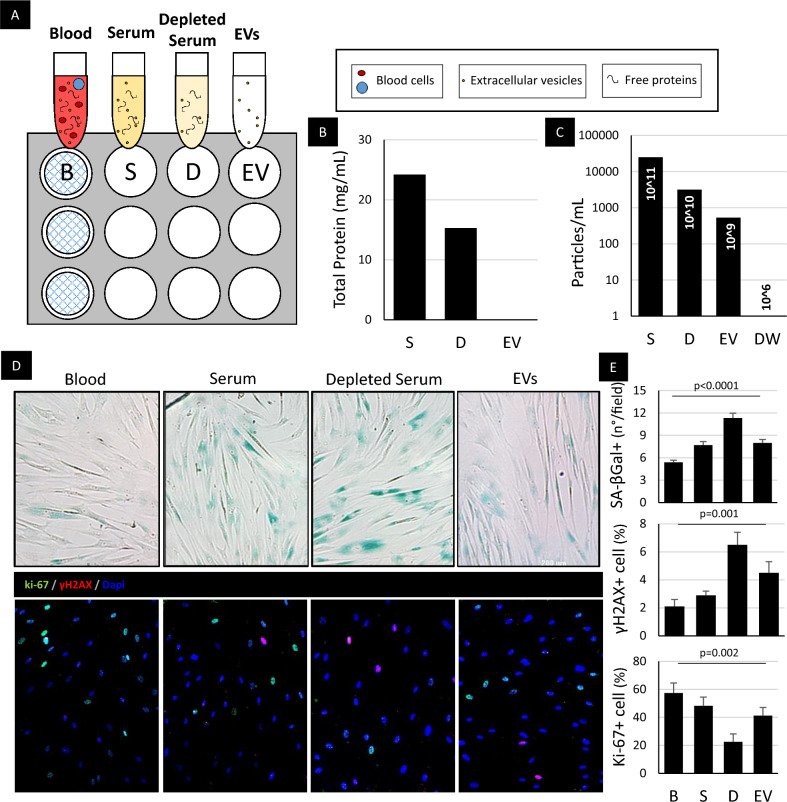
Extracellular vesicles are the main carriers of the rejuvenating effects of neonatal serum. (**A**) Schematic representation of in vitro “heterochronic parabiosis” assay. Cardiac stromal-progenitor cells (CSPCs) obtained from > 55-year old human donors were treated with different fractions of neonatal rat blood: (**B**) whole blood (including all cells), using transwell membranes; S, serum separated from B by centrifugation; (**D**), EV-depleted serum obtained from S by 3-h ultracentrifugation; EV, extracellular vesicles purified from S by density gradient protocol. Volumes of each fraction used for treatment of each well were adjusted to ensure an equal volume of serum in B and S, equal protein concentration in S and D, and equal number and concentration of EVs in S and EV. Each fraction was then diluted in serum-free medium and used to treat CSPC for 72 h. Then CPSCs were washed, fixed, and stained for different senescence markers. (**B**) Total free protein concentration in different blood fractions was measured with the BCA assay. (**C**) EV concentration in different blood fractions measured with Nanoparticle Tracking Analysis. Distilled water (DW) was used as a negative control. **(D**) Representative images of CSPCs after priming with different blood fractions and stained for: senescence-associated β-galactosidase (SA-βGal, blue) – upper panel; proliferation and DNA damage markers ki-67 (green) and γH2AX (red), respectively – lower panel. **(E**) Pooled results of analysis show the highest percentage of senescent cells in CSPCs primed with depleted serum (**D**) where the vesicles’ concentration is the lowest, and recapitulation of total serum’s (S) effect with the fraction of purified EVs. *P*-values were estimated with ANOVA. Data presented are means ± s.e.m. Number of replicates per experiment was three.

Many studies exploring rejuvenation have used young blood (by heterochronic parabiosis, or transfusion), two of which implicated the protein GDF11 as an anti-senescent mediator^28,29^ (conclusions which were later disputed^30,31^;). Since EVs are the soluble effectors of the CDCs^32^, we decided to compare their anti-senescent properties relative to those of other blood fractions in vitro, simulating heterochronic parabiosis model (Methods, Fig. 5A–C). Here, old human donor CSPCs were treated with different fractions of neonatal rat blood. After 48 h’ incubation, CSPCs displayed two indicators of cellular aging (senescence-associated β galactosidase (SA-β-GAL), γH2AX, a DNA damage response marker), and diminished proliferative activity (as reported by Ki-67). Exposure to whole blood or serum decreased SA-β-GAL and γH2AX, while increasing Ki67 positivity. While the EV fraction reproduced these effects, cells treated with EV-depleted serum exhibited the highest degree of senescence, and the least cellular proliferation (Fig. 5D,E). The differences appear to be independent of circulating proteins, insofar as the free protein concentration in the EV-depleted-serum fraction was unchanged relative to total serum, but the concentration of EVs was ~10-times lower. These results implicate EVs as circulating bloodborne endogenous modulators of senescence.

## Discussion

**Figure 6:**
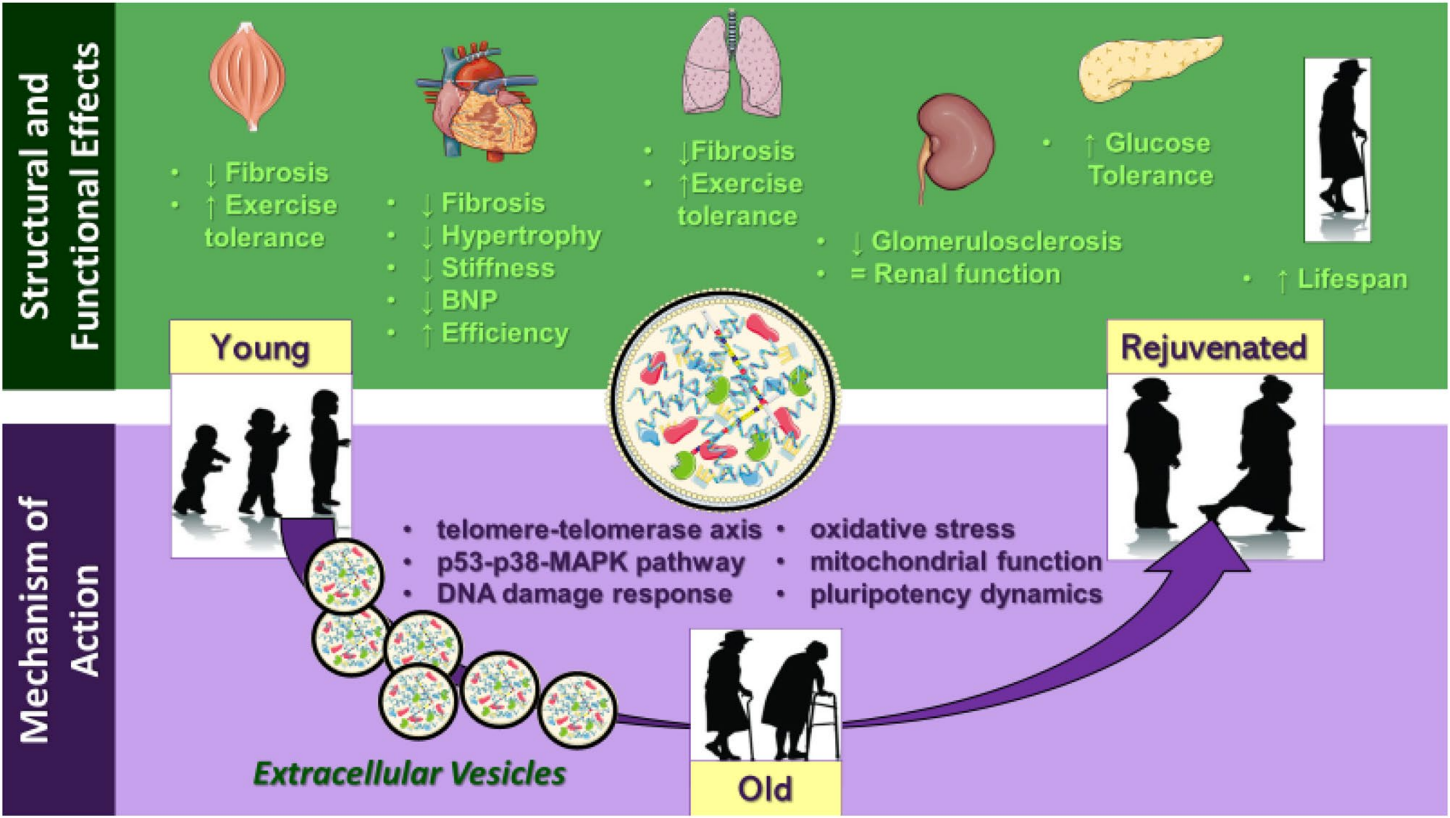
Extracellular vesicles secreted by young cardiac progenitor cells exert broad-ranging anti-aging effects in naturally-aged animals. Given the heterogeneous bioactive content of the vesicles modulation of multiple but synchronized pathways, related with the antisenescence mechanism of action, underlain the observed structural and functional changes.

Repeated systemic administration of young CDC-EVs in aged rodents triggered broad-ranging functional improvements, with concordant structural changes in different organs and associated evidence of tissue rejuvenation (Fig. 6). The beneficial effects of CDC-EVs were maintained over mid-term follow-up, with prolongation of survival of treated animals. But, beyond longevity, the changes we observed in heart and kidney function, glucose metabolism, and exercise tolerance have the potential to improve quality of life, which is an important goal of anti-aging therapies^33^.

Cardiovascular diseases, diabetes, and cancer are aging-related conditions which underlie much morbidity and mortality in the elderly population^1,2,4^. Using a single cell-free therapeutic agent, young CDC-EVs, we demonstrated that all three pathologies can be favorably modulated. Moreover, tissue fibrosis contributing to organ dysfunction^23,34^ was broadly ameliorated (heart, lungs, skeletal muscle and kidneys exhibited less interstitial fibrosis) in CDC-EV treated rats. Based on these findings, CDC-EVs emerge as a strategy capable of targeting pathophysiologic mechanism(s) underlying many age-related chronic conditions. Both, MiR-146 and miR-92a highly enriched in CDC-EVs^11,32^ known to be implicated in aging-related pathways^35,36^ may have played a role in rejuvenating effects observed in our study.

Cellular senescence is thought to contribute to progressive age-related organ dysfunction^4,25^. Senescent cells are characterized by extensive transcriptional changes, telomere attrition, chronically stimulated DNA damage response, mitochondrial dysfunction, the senescence-associated secretory phenotype (SASP), and cell-cycle arrest^37–39^. Previously, we described an anti-senescent effect of young CDC-EVs in vitro^10^. Here, we confirm that cellular rejuvenation, conceived as partial or total reversal of senescence, can be also achieved in vivo in old animals injected with young CDC-EVs. Benefits include telomere elongation in heart cells, less-active DNA damage response (represented by phosphorylated γH2AX,^40^), lower IL-6 levels, and changes in protein levels suggestive of enhanced mitochondrial biogenesis in skeletal muscle. Extensive transcriptomic differences in treated versus control groups were consistent with the observed upregulation of the transcription factor NANOG and extracellular signal-regulated kinase ERK 1/2. Both are recognized regulators and stabilizers of the pluripotency gene regulatory network^15^. Accordingly, we speculate that the mechanism of action of young CDC-EVs is related in part to the control of the dynamic state of pluripotency and reprogramming^41^, a strategy that has been touted in pursuit of rejuvenation^42^. This idea is also supported by the increased self-assembly potential of young CDC-EV-primed senescent human heart cells in vitro.

Among the various approaches being tested to promote rejuvenation^42–45^, the use of young blood or its components has gained particular traction, including trials in humans (^28,46,47^, ClinicalTrials.gov Identifier: NCT02803554). Much effort has been put into identifying the blood component(s) responsible for the rejuvenating effects^47,48^, without conclusive results to date. While acknowledging the limitations of our in vitro “parabiosis” experiment, we observed that EVs are required for the rejuvenating effects of neonatal serum. In fact, treatment with neonatal CDC-EVs in old animals, and in vitro on senescent human cells, induced multiple broad anti-aging effects.

Several limitations of our study need to be considered when interpreting the results. Although we selected a naturally-aged rat model, rodents may do not fully recapitulate the aging process in larger mammals. We did, however, confirm the rejuvenating effects of CDC-EVs in models of human cellular senescence (CSPCs from aged donors, and dermal fibroblasts from progeric patients) in vitro. Additionally, the functional studies were performed sequentially, allowing us to evaluate CDC-EV-induced changes in the same animal, but histopathological evaluation could only be performed at study end-point, so only the differences between groups at that specific time point were assessed. In the in vitro experiment simulating parabiosis, although the EV fraction was purified and separated from free proteins by the density gradient method, we cannot rule out that some individual proteins may be different quantitatively in the EV-depleted serum fraction. Another limitation of the simulated in vitro parabiosis was the lack of true cross-circulation; however, it would have been impossible experimentally, using currently available methods, to deplete EVs selectively in vivo in one paired animal and not the other.

### Translational relevance

Aging-related diastolic dysfunction and the associated heart failure represent unmet clinical needs. Since classical therapeutic targets pursued in systolic heart failure have failed, novel biological interventions are being explored. Here, we find that EVs secreted by young donors’ cells can reverse various aging-related pathologies in senescent rats. Infusion of young CDC-EVs trigger systemic rejuvenation as manifested by improved glucose metabolism, exercise capacity, and survival. In the heart, signs of cellular rejuvenation were associated with structural (less fibrosis and hypertrophy) and functional (decreased stiffness) improvements. Rejuvenating effects of young CDC-EVs were validated in human models of cellular senescence. Given that allogeneic CDCs are already in advanced testing and have proven safe to date, such cells can be used as manufacturing platforms for EVs, enabling rapid progress to clinical testing in a variety of aging-related disorders.

In conclusion, EVs are nanoparticles containing thousands of known and potential signaling molecules (ribonucleic acids, proteins, lipids)^32,49^. EVs can influence multiple pathways synergistically; indeed, our findings indicate or hint that CDC-EVs modulate not only the telomere-telomerase axis^10^ and the p53-p38-MAPK pathway, but also the DNA damage response, oxidative stress, mitochondrial function, and pluripotency dynamics. Even more processes, yet to be explored, may underlie the observed benefits on lifespan, metabolic health, tissue composition and cardiac function. 

## Supplementary Information

Supplementary Information.

Supplementary Figures.

## Data Availability

All data generated or analysed during this study are included in this published article and its supplementary information files.
