# Supplementary material for: Rejuvenating effects of young extracellular vesicles in aged rats and in cellular models of human senescence
Source: Sci Rep. 2023 Jul 28;13:12240. doi: 10.1038/s41598-023-39370-5 (PMC10382547; doi:10.1038/s41598-023-39370-5)
Supplement: Supplementary file 1 — Supplementary Information. [file 41598_2023_39370_MOESM1_ESM.docx]

**SUPPLEMENTAL METHODS**

**Experimental animals**

Twenty-two-month-old Fisher 344 rats (male and female) were obtained from the National Institute of Aging. All animals were studied in accordance with the local guidelines of the Animal Care and Use Committee as published by the National Institute of Health (NIH Publication No. 86-23, revised 1996). Fisher 344 rats were provided with food (Purina rat chow) and water *ad libitum*. Animals were housed in pairs with a dark: light cycle of 12 hours each and an ambient temperature of 22º C.

**Treatment of rats in the main study**

After initial evaluation with echocardiography and exercise testing, a total of 27 animals were divided randomly into two groups: (1) 13 rats treated with extracellular vesicles secreted by

neonatal cardiosphere-derived cells (CDC-EVs), (2) 14 rats receiving phosphate buffered saline (PBS, *i.e.* vehicle control). One rat in the CDC-EV group was excluded from the study because baseline echocardiography showed a dilated left ventricle (LV) and depressed systolic function.

CDCs were grown from 30 freshly-explanted F344 neonatal rat hearts as described (1,2). Briefly, hearts were minced, subjected to enzymatic digestion and then plated on adherent (fibronectin-coated) culture dishes. These explants spontaneously yield monolayer adherent cells (explant-derived cells) which were harvested and plated in suspension culture (10^5^ cells/mL on ultra-low adherence surface) to enable the self-assembly of three-dimensional cardiospheres. Subsequent replating of these cardiospheres on adherent culture dishes yielded CDCs.

CDC-EVs were harvested from passage 2, 8x10^6^ CDCs, from serum-free medium conditioned for 15 days at 90% confluence. Conditioned medium was 0.45 μm filtered to remove cell debris. It was then concentrated with 3 kDA Centricon Plus-70 centrifugal filter, spinning at 3200 xG for 60 minutes at room temperature. The resulting supernatant was precipitated by polyethylene glycol (ExoQuickTC), which yields high quantities of purified EVs (3,4), after overnight incubation at 4ºC. EVs were precipitated by centrifugation at 2000xG for 30 min, resuspended in PBS, and quantified for particle concentration, size and total protein concentration with Bradford assay (Bio-Rad).  CDC-EVs were characterized according to typical EV markers (Suppl. Fig 1a). Particle concentration and size were measured on a NanoSight NS300 (Malvern) in 1:10 diluted samples of CDC-EV containing conditioned medium. The parameters for Nanosight acquisition and analysis were as follows: Camera level: 15; Detection Threshold: 5; Number of videos acquired per sample: 5; Video duration: 30 s (Suppl. Fig 1b). The yield of CDC-EVs was ~2.5x10^10^ particles/10^6^ CDCs. Body weight adjusted dosing was performed based on protein concentration in the EV preparations. We used a higher initial dose of 7.5 μg/g followed by lower monthly doses (1.5 μg/g) of CDC-EVs. CDC-EVs resuspended in 300µL PBS or 300 µL PBS alone were injected percutaneously into the LV cavity under ultrasound guidance.

**Cardiac echocardiography**

Echocardiography was performed at baseline before treatment, and on a monthly basis to assess systolic and diastolic functions (Vevo 3100, Visual Sonics, Toronto, Ontario, Canada), under general anesthesia (Isoflurane 4% for induction followed by 2%) and spontaneous respiration. Two-dimensional long axis and short axis (at the papillary muscle level) LV images were obtained. M-mode tracings were recorded through the anterior and posterior LV walls at the papillary muscle level to measure LV dimension, and LV anterior and posterior wall thickness at end diastole. Pulse-wave Doppler spectra (E and A waves) of mitral inflow were recorded from the apical 4-chamber view, with the sample volume placed near the tips of the mitral leaflets and adjusted to the position at which velocity was maximal and the flow pattern laminar. When the A wave could be clearly identified, E/A ratio was used to assess diastolic function as described. Systolic function was assessed by LV ejection fraction (LVEF) and fractional area change (FAC) calculated from the short axis view. Tissue Doppler imaging was used to obtain the velocity of the early diastolic E’ wave at the septal mitral annulus. Right ventricular function was assessed with tricuspid annular plane systolic excursion (TAPSE).

**Exercise test**

Rats were acclimated to the treadmill by walking at a speed of 5 m/min for 5 minutes before each test, on a 3-lane Columbus Instruments treadmill. The protocol for the maximal exercise capacity test consisted of warming up at 5 m/min for 5 minutes followed by 3 m/min-increases in speed every 3 minutes until the rat reached exhaustion. Rats were considered exhausted when they failed to stay off of the shock grid. The grade of the treadmill was set to 15° for the duration of the test.

**Hemodynamic measurements**

Hemodynamic measurements were performed at endpoint. Under general anesthesia (Isoflurane 4-5% for induction followed by 2%), rats were intubated and maintained under controlled respiration. Left thoracotomy was performed and the heart apex was exposed. A 2F conductance catheter (SPR-838, Millar, Houston, Texas, USA) was then introduced into the LV cavity using a transapical approach. Once inside of the LV cavity and after recording stability was reached, the end systolic and end diastolic pressures and volumes were recorded. Data for determination of LV end-diastolic and end-systolic pressure-volume relationships (EDPVR and ESPVR, respectively) were obtained by temporary inferior vena cava occlusion. The time constant of isovolumetric LV pressure fall (Tau) was calculated as described. All data were collected and analyzed using pressure-volume analysis software (LabChart, ADInstruments, Colorado Springs, Colorado, USA).

**Glucose metabolism study**

In a separate group of 11 male and female 22-month-old Fisher 344 rats, glucose metabolism was evaluated with the glucose tolerance test (GTT). After an 8-hour fasting period, baseline (min 0 of GTT) blood samples were collected to determine insulin and glucose levels, followed by 10% glucose solution administration via intraperitoneal (IP) injection. After 30 minutes, another blood sample was collected for insulin and glucose measurements and afterwards, consecutive glucose levels were checked every 30 minutes up to 2 hours with test strips *via* tail vein. Four weeks after the initial GTT, rats were randomly allocated to receive an IP dose of CDC-EVs (7.5 μg/g) resuspended in PBS (n=5) or PBS alone (n=6). Forty-eight hours later, the GTT was repeated.

**Tissue and blood collection**

Blood samples (1 mL) were collected early in the morning at baseline and on a monthly basis via external jugular vein puncture. Serum was separated, aliquoted and sent to an external lab for analyses and/or frozen at -80 °C. At endpoint, complete blood counts were analyzed. At study end, hearts were arrested in diastole (intra-ventricular injection of KCl) and excised. Other organs and tissues were collected at the same time. For histology, organ slices were embedded in OCT compound (Sakura Finetek, Torrance, California, USA) and frozen at -80 ºC. For protein and RNA quantification, tissue samples were maintained in RNA and protein stabilization reagent (Allprotect, Qiagen, Venlo, Netherlands) and frozen at -80 ºC.

**Histology**

***Collagen content quantification***

Heart, skeletal muscle, and lung tissues were cut in 5 μm sections, with kidneys cut in 2 μm sections. Histochemistry included Masson’s Trichrome stain for determination of fibrosis and overall tissue damage. Masson’s Trichrome stained slides were quantified using a Zeiss bright-field microscope interfaced with an analog camera, and ImageJ (NIH) bioquantification software, expressing the collagen content as a percentage of the area of each segment (8-10 images/slide). The assessment and analysis of the data was carried out in a blinded fashion by two independent pathologists. Prior to acquisition, the camera was white-balanced to ensure uniform background color was maintained, and the microscope’s light intensity was maintained at a constant level to ensure background values were similar for each acquired image. Likewise, the f-stop for the camera was maintained at a constant level for each acquired image.

***Measurement of cross-sectional area***

Heart tissue was embedded in OCT compound and 5 µm cryosections were stained with H&E. Light micrographs were taken using an Olympus BX43F microscope with CellSens software, and myocytes were measured by tracing in ImageJ. Myocytes with central nuclei and intact cell borders that were in cross-section (i.e., round and not oval) were chosen for analysis. For each rat, a total of 40 to 45 myocytes were circled from 9 to 11 micrographs taken from different sections to ensure that multiple areas were sampled. These area measurements were converted to µm^2^ using the conversion rate provided by the CellSens software (273.81 nm=1 pixel).

***Immunostaining*** ***and cytokine expression in heart tissue***

For detection of senescence markers in the heart tissue, OCT-cut tissue sections were fixed with 4% PFA and stained with the following primary antibodies for confocal microscopy: α-sarcomeric actinin (Abcam 72592) and γH2AX (Abcam 11174). The appropriate fluorescently-conjugated secondary antibodies (Invitrogen) were applied and all slides were counterstained for DAPI (Molecular Probes). Five to 10 images per section or slide were imaged at x20 magnification using a confocal laser microscope and analyzed using Image-J (NIH) bioquantification software.

IL-6 levels were measured in mildly homogenized heart tissue protein lysates with MyBioSource rat ELISA kit (MBS355410) and adjusted for the total protein concentration quantified by the Pierce™ BCA Protein Assay Kit (ThermoFisher Scientific 23250).

***Telomere Length Assay***

To measure telomere length, the hearts from each treatment group were fixed in 4% paraformaldehyde and then frozen in OCT compound (Tissue-Tek) for cryosectioning. 5 µm sections were cut via cryotome by the Cedars-Sinai Pathology Core and mounted onto glass slides. The cardiac tissue was permeabilized and telomeres were stained using Fluorescent In Situ Hybridization (Telomere PNA FISH Kit/Cy3; DAKO). Rabbit primary antibodies raised against rat sarcomeric α-actinin (Abcam 72592) and goat anti-rabbit FITC-conjugated secondary antibodies (Abcam) were used to identify cardiomyocytes. DAPI was used as a nuclear stain. 100x images (20-40 images per animal) were taken using the BIOREV Keyance BZ-9000 fluorescent microscope, under identical imaging settings between slides. Telomere length was analyzed using ImageJ (NIH), by measuring the integrated optical density of the Cy3-channel within the nuclear borders, running perpendicular to the image plane after subtracting the background and adjusting to the nuclei area.

**Immunoblot**

Ice-cold RIPA lysis buffer (Thermo Scientific) supplemented with a protease/phosphatase inhibitor cocktail and EDTA (Thermo Scientific) was added to the tissue which was then homogenized using an electric homogenizer and processed for immunoblotting. Briefly, the homogenate was centrifuged at 14,000 g for 30 minutes at 4°C and the pellet was discarded. Total protein concentration of the supernatant was measured using a bicinchoninic acid (BCA) assay (Thermo Scientific). Tissue homogenates were diluted using a 4X LDS sample buffer, 10X reducing agent, and normalized to volume with RIPA lysis buffer. Thirty µg of protein was resolved on a 4-12% Bis-Tris sodium dodecyl sulfate (SDS) polyacrylamide gel (Novex) at 130 V for 60 minutes. Proteins were then immobilized on a PVDF (Thermo Scientific) membrane using a wet transfer module (Bio-Rad) at 300 mA for 1.5 h. Membranes were incubated in Ponceau S and visualized to ensure equal loading. Membranes were then blocked for 1 h at room temperature in 5% (w/v) non-fat dried milk (Bio-Rad) dissolved in tris-buffered saline with 0.05% Tween-20 (TBS-T; Thermo Scientific) on a rocker. Following the 1 h block, membranes were then incubated overnight with primary antibodies diluted in blocking solution at 4°C with shaking. Primary antibodies are as follows: Citrate Synthase (1:1000, Abcam ab96600) and Total OXPHOS (1:1000, Abcam MS604-300). Following the overnight incubation, membranes were washed with TBS-T (3 x 5 minutes) and then probed with the appropriate horseradish peroxidase conjugated secondary antibody for 2 h at room temperature. After the secondary incubation, membranes were washed as previously described and incubated in an enhanced chemiluminescent solution (Thermo Scientific) and immunoreactivity was visualized using a ChemiDoc (Bio-Rad). Protein abundance was analyzed by densitometry using Image Lab v6.0 software (Bio-Rad) and proteins of interest were normalized for loading against Ponceau S stained bands.

**Serum markers**

Serum was analyzed externally by Antech diagnostic laboratory (Los Angeles). Serum levels of BNP and insulin were analyzed by commercially available rat ELISA kits (MyBioSource, Inc. San Diego, CA, USA). The number of animals in each group and the time points when the samples were analyzed are specified on each figure.

**RNA isolation, sequencing, and bioinformatic analysis**

RNA was isolated from heart samples with the RNeasy kit (Qiagen, Venlo, Netherlands). RNA sequencing and the 2-Dimensional hierarchical clustering Nugen Ovation RNA-Seq System V2 kit was used to generate double-stranded cDNA using a mixture of random and poly (T) priming. Kapa LTP library kit (Kapa Biosystems, Wilmington MA) was used to make the sequencing library. The workflow consists of fragmentation of double stranded cDNA, end repair to generate blunt ends, A-tailing, adaptor ligation and PCR amplification. Different adaptors were used for multiplexing samples in one lane. Sequencing was performed on Illumina HiSeq 2500 for a pair read 100 run. Data quality check was done on Illumina SAV. Demultiplexing was performed with Illumina CASAVA 1.8.2. The reads were first mapped to the latest UCSC transcript set using Bowtie2 version 2.1.0 and the gene expression level was estimated using RSEM v1.2.15 (Accession number: GSE85888). TMM (trimmed mean of M-values) was used to normalize the gene expression. Differentially expressed genes were identified using the edgeR program. Genes showing altered expression with p<0.05 and more than 2 fold changes were considered differentially expressed (Suppl 3, Suppl 5, Suppl 9). The pathway and network analyses were performed using Ingenuity pathway analysis (IPA). IPA computes a score for each network according to the fit of the set of supplied focus genes. These scores indicate the likelihood of focus genes to belong to a network versus those obtained by chance. A score > 2 indicates ~99% confidence that a focus gene network was not generated by chance alone. The canonical pathways generated by IPA are the most significant for the uploaded data set. Fischer’s exact test with FDR option was used to calculate the significance of the canonical pathway. 2-Dimensional hierarchical clustering used genes with at least 2 times fold change difference (log2) between PBS and CDC-EV groups. Each column represents a rat analyzed heart tissue and each row a gene. Probe set signal values were normalized to the mean across rat analyzed tissues. The relative level of gene expression is depicted from the lowest (green) to the highest (red), according to the scale shown; examples of fold changes of transcripts for genes involved in the various pathways of interest are plotted.

**Effect on abdominal fat**

Once the animals were euthanized, the abdominal cavity was completely exposed and pictures of the viscera were taken in all rats. Visceral fat was analyzed by measuring the area of fat pads and expressed as a percentage of the total abdominal cavity area using Image J software.

***In vitro* studies**

All protocols were approved by the institutional review board for human subject research.

***Human heart cell isolation and culturing***

When minced human heart tissue is grown in primary culture, it spontaneously gives rise to monolayers of cardiac stromal and progenitor cells (CSPCs). In brief, myocardial biopsies from hearts of deceased tissue donors were minced into small fragments, digested with collagenase, and cultured on fibronectin-coated dishes. CSPCs reached confluence by 2–3 weeks, at which time they were harvested using 0.25% trypsin (GIBCO), purified from tissue and cell debris, and re-plated as needed. Cultures were maintained in 5% CO_2_ at 37°C, using IMDM basal medium (GIBCO) supplemented with 20% FBS (Hyclone), 1% penicillin/streptomycin (Invitrogen; 15140-122), and 0.1 ml 2-mercaptoethanol.

***Progeric dermal fibroblasts***

Dermal fibroblasts from progeric patients were acquired from the Progeria Research Foundation and cultured per protocol in DMEM (Invitrogen; 11960-044, high glucose without L-glutamine) supplemented with 15% FBS (Hyclone), 1% (1X) Penicillin-Streptomycin (Invitrogen; 15140-122) and 1% (1X) L-glutamine (Invitrogen; 25030-081) 200mM.

***Isolation and characterization CDC-derived extracellular vesicles***

CDC-EVs were harvested from young (pediatric <2-years old donors) CDCs at passage 4, from serum-free medium conditioned by CDCs for 15 days at 90% confluence. We used the same procedure as for rat CDC-EVs to purify human CDC-EVs. Total protein concentration-adjusted doses of CDC-EVs resuspended in serum-free medium were used to prime old (> 55-year old) human donors CSPCs and progeric fibroblasts for 48 to 72 hours.

***RT-PCR Array***

RNA was extracted from CSPCs and progeric fibroblasts using RNeasy Plus mini kit (QIAGEN). For further purification of RNA, the RNase-Free DNase set (Qiagen) was used, and the RNA purity and concentration were assessed by Nanodrop. cDNA was generated from the purified RNA using the RT2 First Strand kit (Qiagen). PCR was performed on an Applied Biosystems 96-well fast block cycler using RT2 Profiler PCR Array human Telomeres & Telomerase (Qiagen 330231, PAHS-010Z) and RT2 Real-Time SYBR Green PCR Master Mix (Qiagen). The array evaluated the expression of 84 genes and five housekeeping genes for data normalization. Gene expression was then amplified over the course of 40 cycles and analyzed by ΔΔCt method.

***Proteome profiler assay***

The Proteome Profiler Human Cell Stress Array Kit (ARY018; R&D Systems) was used. CSPCs were lysed using proteome profiler lysis buffer 17 and incubated overnight with Human Phospho-Kinase Array nitrocellulose membranes which were spotted with different capture and control antibodies. Unbound proteins were washed away, and the array was then incubated with a cocktail of biotinylated detection antibodies. After application of Streptavidin-HRP and chemiluminescent detection reagents, a signal was produced at each capture spot based on the amount of bound phosphorylated protein. The average signal (pixel density) of the pair of duplicate spots representing each protein was determined after subtracting an averaged background signal.

***Immunohistochemistry***

Cells were washed with PBS and fixed with 4% paraformaldehyde followed by blocking solution (Dako). Cells were incubated overnight at 4°C with the primary antibody, washed 3 times with PBS, and incubated with a secondary antibody of the appropriate species. The microscope analyses were performed using an epifluorescence microscope (Zeiss). Antibodies used were as follows: anti-beta Actin (Abcam 6277), anti-H2AX (Abcam 11174), and anti-ki-67 (Abcam 15580). Secondary antibodies used were goat anti-mouse IgG secondary antibody, Alexa Fluor® 488 conjugate (1:500, Thermo Fisher Scientific), donkey anti-rabbit IgG secondary antibody Alexa Fluor 546 (1:500, Thermo Fisher Scientific), and in situ cell death detection kit, fluorescein (Roche). Nuclei were identified with DAPI (Sigma).

***Self-assembly (cardiosphere formation) test***

Plated at the same number on day 0, heart explant-derived cells from an old human donor were treated with young CDC-EVs or serum-free medium (control group) on day 1. After 4 days, cells were detached and counted, then resuspended at a density of 3*10^4cells/ml in serum-free medium on ultra-low attachment dishes. Newly formed cardiosphere (CSp) concentration and size were measured after 72 hours using the Beckman Multisizer 4. First the CSp medium was analyzed for background subtraction. Then the diluted CSp suspension was analyzed. The pulses were converted to sizes ranging from 20.2 to 336 um, and the particle count between 40 and 300 μm was determined; objects in this range were considered to be CSp, whereas objects 20-40 μm were considered to be single cells. CSp number was calculated by multiplying by volume x dilution factor.

***Population-doubling time***

Total number of viable cells before plating and after the experimental time was calculated with the TC20™ Automated Cell Counter (Bio-Rad). Cell proliferation was estimated with population doubling time (PDT), according to the formula: PDT = duration of culture (hours) x log (2)/ log (final number of cells) – log (initial number of cells) (Roth V. 2006 Doubling Time Computing, Available from: http://www.doubling-time.com/compute.php).

***Detection of senescent cells***

Senescent cells were detected by the presence of senescence-associated β-galactosidase activity (SA-β-GAL; Abcam). When cell density was high and cells borders weren’t clearly identifiable, SA-β-GAL positive areas were quantified with Image J; when the density was lower and the cells were non-confluent, the positive cell number/optical field was reported.

***In vitro simulation of “ heterochronic parabiosis” experiment and EVs iodixanol gradient purification***

In this assay, old human donor CSPCs were treated with different fractions of neonatal rat blood. Neonatal blood was collected from male and female pups (0-2 days old) and mixed together to ensure an adequate volume for the planned experiments. Four different blood fractions were used in the consecutive experiments: total blood with cells (B), serum (S), EVs-depleted serum (D) and purified EVs. In all cases, blood fractions were resuspended in serum-free medium to prime old human donors CSPCs. The total blood fraction was used with transwell membranes to avoid contamination of the CSPCs with blood cells. Serum was separated from floating cells and cell debris by low speed centrifugation at 2,500 x g for 30 min, 4 °C. For the EVs-depleted fraction, serum was subjected to two consecutive rounds of high-speed ultracentrifugation (100,000 x g, 90 min, 4 °C, k-Factor= 245.5) and the supernatant was collected for the experiments. Purified EVs were isolated from the pellet collected after the first round of ultracentrifugation (described above), resuspended in 0.22 µm-filtered PBS. To ensure the separation of the EVs from other particulate material such as extracellular protein complexes, the EVs were submitted to iodixanol density gradient purification. To do so, pelleted EVs containing 30% (wt/vol, 1.20 g/mL) iodixanol were allowed to float (bottom to top) into a 4-layer (F1:F4) discontinuous density gradient (F1: 1.18 g/mL; F2: 1.15 g/mL; F3: 1.10 g/mL; F4: 1.08 g/mL) for 3h 50 min at 100,000 x g (4 ºC). EVs are known to float at 1.10-1.15 g/mL (5), whereas protein complexes remain in the dense fractions at the bottom of the tube. F2 and F3 density fractions were then pooled and washed in PBS (100,000 x g, 60 min, 4ºC) for the recovery of pure EVs.

All fractions (except total blood) were then subjected to total protein concentration quantification by the Pierce™ BCA Protein Assay Kit (ThermoFisher Scientific 23250) and the number of particles was measured with Nanoparticle Tracking Analysis. To ensure bioequivalence of the used doses, the volume of each fraction used for treatment of each well were adjusted to ensure an equal volume of serum in B and S, equal protein concentration in S and D, and equal number and concentration of EVs in S and EV. Each fraction was then diluted in serum-free medium and used to treat CSPC for 72 hours. Then CPSCs were washed, fixed, and stained for different senescence markers.

**Statistical Analysis**

The main end-point of the study reflected functional improvement of the heart: E/E’ ratio. Based on our previous results (~10 ± 5% of differences in the functional tests between experimental groups), a sample size of ~9-10 animals per group (assuming a 5% significance level and an 80% power level) was estimated, using a statistical software program (GB-Stat Version 10.0, Dynamic Microsystems Inc). Assuming a 20% age-related mortality in these old rats and 10% peri-procedural mortality, observed in our lab, we acquired 30 animals for our experimental groups. Three rats died before starting the study, the remaining 27 were allocated to receive CDC-EVs (n=13) or PBS (n=14). One rat from the CDC-EV was excluded after detecting that on baseline echocardiography left ventricular ejection fraction was depressed, so was not considered for the final analysis. For the histological evaluations, we based our estimation on previous results with CDCs on fibrosis (1,6,7). The anticipated standard deviation was 2.4% and the anticipated magnitude of the difference was 4.5%. Thus, the estimated sample size was of ~5-6 per group, for an assessment by student's T test with an alpha value of 0.05 and beta value of at least 0.8. Since 7 rats per group survived after 4-month treatment period, whenever possible we used all animals’ samples for postmortem analysis. Samples if excluded was because of quality (sample, imaging, other type of analysis) reasons. For next generation RNA sequencing of heart tissue, we randomly selected three animals per group limited by the expensive costs of this test. All results are presented as mean ± SD (± SEM in figures) or percentages, for continuous and categorical variables, respectively. Significance of differences was assessed by Student t test or with 1-way ANOVA in case of multiple groups if the distribution of the variable was normal; otherwise, the Mann-Whitney or Kruskal-Wallis tests were used. Paired t-test was used to determine significance between baseline and endpoint in the same group of animals. All probability values reported are 2-sided, with p<0.05 considered significant. IBM SPSS Statistics 20 was used for all analyses. For in vitro studies the lowest number of replicates per experiment was three. For in vitro RT-PCR Array, gene expression was analyzed online, using QIAGEN data analysis center (http://www.qiagen.com/us/shop/genes-and-pathways/data-analysis-center-overview-page/rt2-profiler-pcr-arrays-data-analysis-center). To minimize the potential noise introduced by measurements below detection threshold, mRNAs with Ct value > 35 in all groups were considered as undetected. Specifically, the expression levels of mRNAs were evaluated by a comparative Ct method using median of expressed housekeeping mRNAs for normalization. The data were only used if the output passed the quality control test with respect to array genomic DNA contamination, reproducibility and reverse transcriptase efficiency. Fold-change calculations or gene expression ratios were calculated using the classic, well-established, and widely adopted ∆∆CT method. The p-values were calculated using a Student’s t-test (two-tail distribution and equal variances between the two samples) on the replicate 2–ΔCT values for each gene in each group (CDC-EVs) compared to the control group (PBS). The p-values less than 0.05 were indicated as significant. Each sample was used in duplicate or triplicate for validation purposes.

**Supplementary References**

1. Smith RR, Barile L, Cho HC, Leppo MK, Hare JM, Messina E, Giacomello A, Abraham MR, Marbán E. Regenerative potential of cardiosphere-derived cells expanded from percutaneous endomyocardial biopsy specimens. Circulation. 2007 Feb 20;115(7):896-908. Epub 2007 Feb 5.

2. Grigorian-Shamagian L, Liu W, Fereydooni S, Middleton RC, Valle J, Cho JH, Marbán E. Cardiac and systemic rejuvenation after cardiosphere-derived cell therapy in senescent rats. Eur Heart J. 2017 Oct 14;38(39):2957-2967. doi: 10.1093/eurheartj/ehx454.

3. Rider MA, Hurwitz SN, Meckes DG Jr. ExtraPEG: A Polyethylene Glycol-Based Method for Enrichment of Extracellular Vesicles. Sci Rep. 2016 Apr 12;6:23978. doi: 10.1038/srep23978.

4. Taylor DD, Shah S. Methods of isolating extracellular vesicles impact down-stream analyses of their cargoes. Methods. 2015 Oct 1;87:3-10. doi: 10.1016/j.ymeth.2015.02.019.

5. Kowal, J., Arras, G., Colombo, M., Jouve, M., et al., Proteomic comparison defines novel markers to characterize heterogeneous populations of extracellular vesicle subtypes. Proc. Natl. Acad. Sci. 2016, 113, 968–977.

6. Tseliou E, de Couto G, Terrovitis J, Sun B, Weixin L, Marban L, Marban E. Angiogenesis, cardiomyocyte proliferation and anti-fibrotic effects underlie structural preservation postinfarction by intramyocardially-injected cardiospheres. PLoS ONE 2014;9:e88590.

7. Aminzadeh MA, Tseliou E, Sun B, Cheng K, Malliaras K, Makkar RR, Marbán E. Therapeutic efficacy of cardiosphere-derived cells in a transgenic mouse model of non-ischaemic dilated cardiomyopathy. Eur Heart J 2015;36:751-62.

Suppl Table 1. Differentially expressed genes in cardiac tissue of CDC-EV (group 2) and control PBS (group 1) rats. Only genes being differentially expressed with a fold change of at least 1.5 and a p-value < 0.05 are shown.

Suppl Table 2. All detectable genes in cardiac tissue of CDC-EV (group 2) and control PBS (group 1) rats.
