# Supplementary material for: Rejuvenating effects of young extracellular vesicles in aged rats and in cellular models of human senescence
Source: Sci Rep. 2023 Jul 28;13:12240. doi: 10.1038/s41598-023-39370-5 (PMC10382547; doi:10.1038/s41598-023-39370-5)
Supplement: Supplementary file 2 — Supplementary Figures. [file 41598_2023_39370_MOESM2_ESM.pptx]

## Slide 1
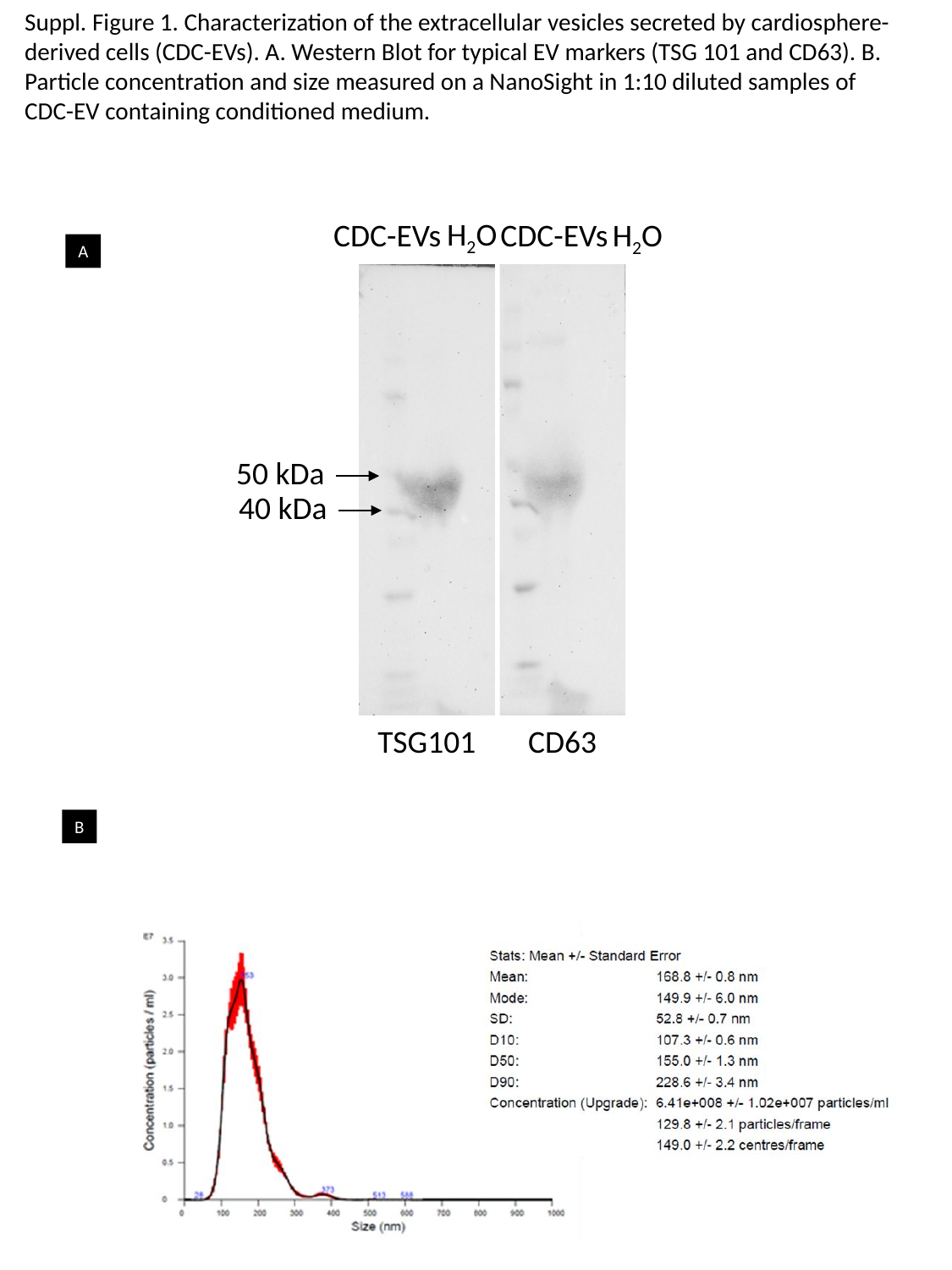

Suppl. Figure 1. Characterization of the extracellular vesicles secreted by cardiosphere-derived cells (CDC-EVs). A. Western Blot for typical EV markers (TSG 101 and CD63). B. Particle concentration and size measured on a NanoSight in 1:10 diluted samples of CDC-EV containing conditioned medium.
H2O
CDC-EVs
CDC-EVs
H2O
50 kDa
40 kDa
TSG101
CD63
A
B

## Slide 2
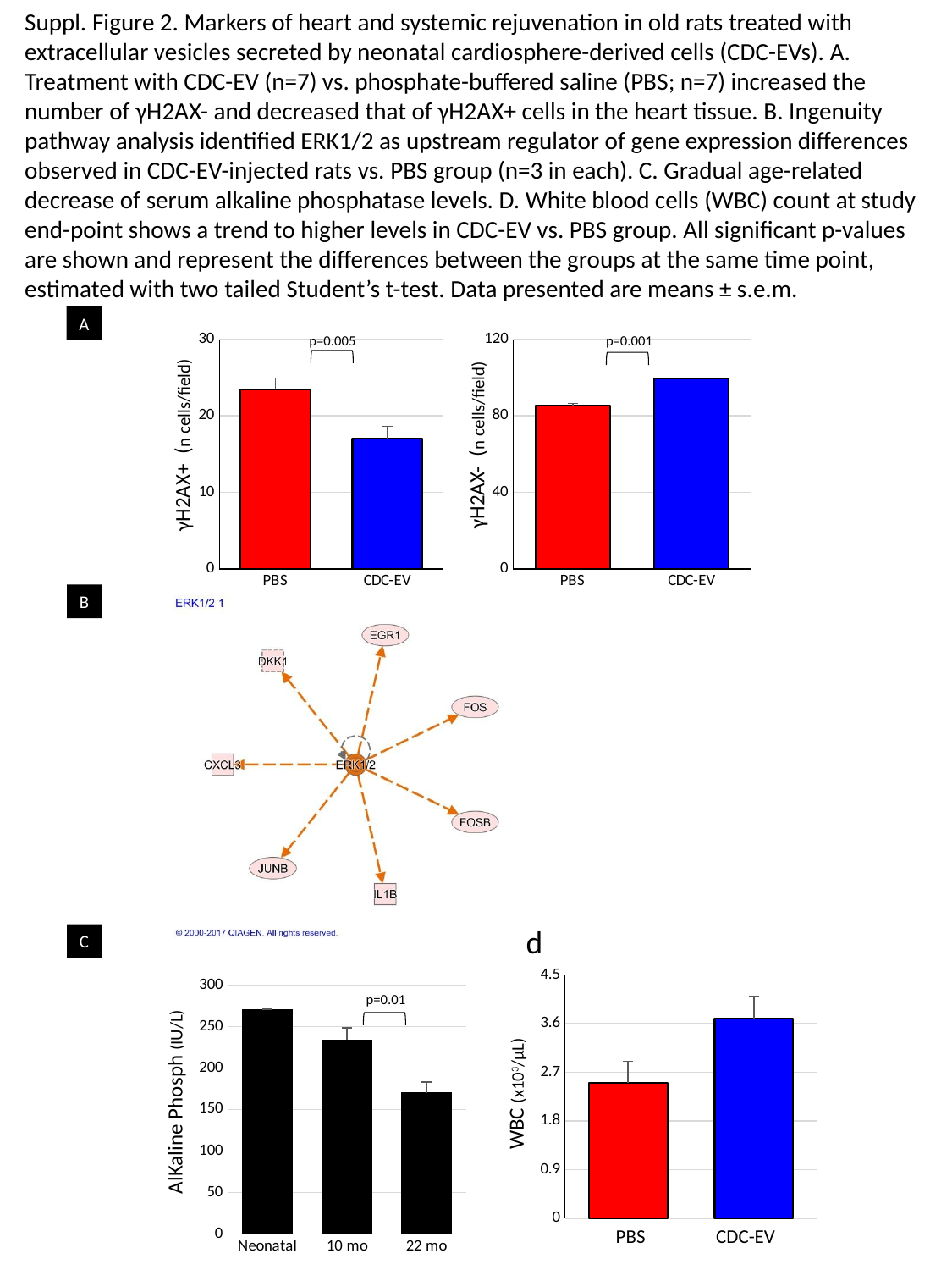

Suppl. Figure 2. Markers of heart and systemic rejuvenation in old rats treated with extracellular vesicles secreted by neonatal cardiosphere-derived cells (CDC-EVs). A. Treatment with CDC-EV (n=7) vs. phosphate-buffered saline (PBS; n=7) increased the number of γH2AX- and decreased that of γH2AX+ cells in the heart tissue. B. Ingenuity pathway analysis identified ERK1/2 as upstream regulator of gene expression differences observed in CDC-EV-injected rats vs. PBS group (n=3 in each). C. Gradual age-related decrease of serum alkaline phosphatase levels. D. White blood cells (WBC) count at study end-point shows a trend to higher levels in CDC-EV vs. PBS group. All significant p-values are shown and represent the differences between the groups at the same time point, estimated with two tailed Student’s t-test. Data presented are means ± s.e.m.
A
p=0.001
### Chart
| Category | |
|---|---|
| PBS | 23.4 |
| CDC-EV | 17.0 |p=0.005
### Chart
| Category | |
|---|---|
| PBS | 85.4 |
| CDC-EV | 99.4 |
γH2AX- (n cells/field)
γH2AX+ (n cells/field)
B
d
C
### Chart
| Category | 16 weeks |
|---|---|
| PBS | 2.5 |
| Neo-EV | 3.7 |
### Chart
| Category | |
|---|---|
| Neonatal | 270.0 |
| 10 mo | 234.0 |
| 22 mo | 170.2 |p=0.01
WBC (x103/µL)
AlKaline Phosph (IU/L)
PBS CDC-EV

## Slide 3
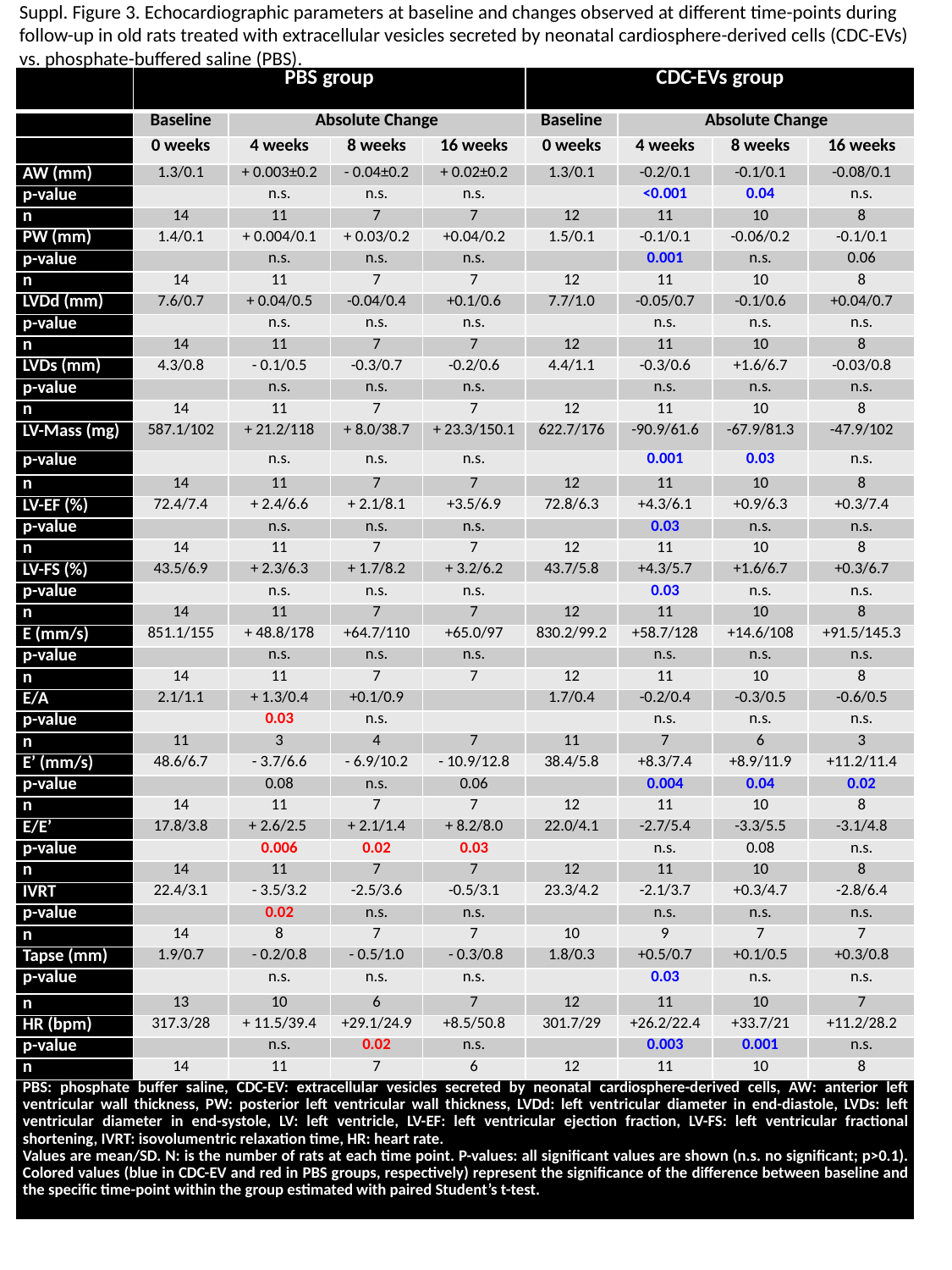

Suppl. Figure 3. Echocardiographic parameters at baseline and changes observed at different time-points during follow-up in old rats treated with extracellular vesicles secreted by neonatal cardiosphere-derived cells (CDC-EVs) vs. phosphate-buffered saline (PBS).
| | PBS group | | | | CDC-EVs group | | | |
| --- | --- | --- | --- | --- | --- | --- | --- | --- |
| | Baseline | Absolute Change | | | Baseline | Absolute Change | | |
| | 0 weeks | 4 weeks | 8 weeks | 16 weeks | 0 weeks | 4 weeks | 8 weeks | 16 weeks |
| AW (mm) | 1.3/0.1 | + 0.003±0.2 | - 0.04±0.2 | + 0.02±0.2 | 1.3/0.1 | -0.2/0.1 | -0.1/0.1 | -0.08/0.1 |
| p-value | | n.s. | n.s. | n.s. | | <0.001 | 0.04 | n.s. |
| n | 14 | 11 | 7 | 7 | 12 | 11 | 10 | 8 |
| PW (mm) | 1.4/0.1 | + 0.004/0.1 | + 0.03/0.2 | +0.04/0.2 | 1.5/0.1 | -0.1/0.1 | -0.06/0.2 | -0.1/0.1 |
| p-value | | n.s. | n.s. | n.s. | | 0.001 | n.s. | 0.06 |
| n | 14 | 11 | 7 | 7 | 12 | 11 | 10 | 8 |
| LVDd (mm) | 7.6/0.7 | + 0.04/0.5 | -0.04/0.4 | +0.1/0.6 | 7.7/1.0 | -0.05/0.7 | -0.1/0.6 | +0.04/0.7 |
| p-value | | n.s. | n.s. | n.s. | | n.s. | n.s. | n.s. |
| n | 14 | 11 | 7 | 7 | 12 | 11 | 10 | 8 |
| LVDs (mm) | 4.3/0.8 | - 0.1/0.5 | -0.3/0.7 | -0.2/0.6 | 4.4/1.1 | -0.3/0.6 | +1.6/6.7 | -0.03/0.8 |
| p-value | | n.s. | n.s. | n.s. | | n.s. | n.s. | n.s. |
| n | 14 | 11 | 7 | 7 | 12 | 11 | 10 | 8 |
| LV-Mass (mg) | 587.1/102 | + 21.2/118 | + 8.0/38.7 | + 23.3/150.1 | 622.7/176 | -90.9/61.6 | -67.9/81.3 | -47.9/102 |
| p-value | | n.s. | n.s. | n.s. | | 0.001 | 0.03 | n.s. |
| n | 14 | 11 | 7 | 7 | 12 | 11 | 10 | 8 |
| LV-EF (%) | 72.4/7.4 | + 2.4/6.6 | + 2.1/8.1 | +3.5/6.9 | 72.8/6.3 | +4.3/6.1 | +0.9/6.3 | +0.3/7.4 |
| p-value | | n.s. | n.s. | n.s. | | 0.03 | n.s. | n.s. |
| n | 14 | 11 | 7 | 7 | 12 | 11 | 10 | 8 |
| LV-FS (%) | 43.5/6.9 | + 2.3/6.3 | + 1.7/8.2 | + 3.2/6.2 | 43.7/5.8 | +4.3/5.7 | +1.6/6.7 | +0.3/6.7 |
| p-value | | n.s. | n.s. | n.s. | | 0.03 | n.s. | n.s. |
| n | 14 | 11 | 7 | 7 | 12 | 11 | 10 | 8 |
| E (mm/s) | 851.1/155 | + 48.8/178 | +64.7/110 | +65.0/97 | 830.2/99.2 | +58.7/128 | +14.6/108 | +91.5/145.3 |
| p-value | | n.s. | n.s. | n.s. | | n.s. | n.s. | n.s. |
| n | 14 | 11 | 7 | 7 | 12 | 11 | 10 | 8 |
| E/A | 2.1/1.1 | + 1.3/0.4 | +0.1/0.9 | | 1.7/0.4 | -0.2/0.4 | -0.3/0.5 | -0.6/0.5 |
| p-value | | 0.03 | n.s. | | | n.s. | n.s. | n.s. |
| n | 11 | 3 | 4 | 7 | 11 | 7 | 6 | 3 |
| E’ (mm/s) | 48.6/6.7 | - 3.7/6.6 | - 6.9/10.2 | - 10.9/12.8 | 38.4/5.8 | +8.3/7.4 | +8.9/11.9 | +11.2/11.4 |
| p-value | | 0.08 | n.s. | 0.06 | | 0.004 | 0.04 | 0.02 |
| n | 14 | 11 | 7 | 7 | 12 | 11 | 10 | 8 |
| E/E’ | 17.8/3.8 | + 2.6/2.5 | + 2.1/1.4 | + 8.2/8.0 | 22.0/4.1 | -2.7/5.4 | -3.3/5.5 | -3.1/4.8 |
| p-value | | 0.006 | 0.02 | 0.03 | | n.s. | 0.08 | n.s. |
| n | 14 | 11 | 7 | 7 | 12 | 11 | 10 | 8 |
| IVRT | 22.4/3.1 | - 3.5/3.2 | -2.5/3.6 | -0.5/3.1 | 23.3/4.2 | -2.1/3.7 | +0.3/4.7 | -2.8/6.4 |
| p-value | | 0.02 | n.s. | n.s. | | n.s. | n.s. | n.s. |
| n | 14 | 8 | 7 | 7 | 10 | 9 | 7 | 7 |
| Tapse (mm) | 1.9/0.7 | - 0.2/0.8 | - 0.5/1.0 | - 0.3/0.8 | 1.8/0.3 | +0.5/0.7 | +0.1/0.5 | +0.3/0.8 |
| p-value | | n.s. | n.s. | n.s. | | 0.03 | n.s. | n.s. |
| n | 13 | 10 | 6 | 7 | 12 | 11 | 10 | 7 |
| HR (bpm) | 317.3/28 | + 11.5/39.4 | +29.1/24.9 | +8.5/50.8 | 301.7/29 | +26.2/22.4 | +33.7/21 | +11.2/28.2 |
| p-value | | n.s. | 0.02 | n.s. | | 0.003 | 0.001 | n.s. |
| n | 14 | 11 | 7 | 6 | 12 | 11 | 10 | 8 |
| PBS: phosphate buffer saline, CDC-EV: extracellular vesicles secreted by neonatal cardiosphere-derived cells, AW: anterior left ventricular wall thickness, PW: posterior left ventricular wall thickness, LVDd: left ventricular diameter in end-diastole, LVDs: left ventricular diameter in end-systole, LV: left ventricle, LV-EF: left ventricular ejection fraction, LV-FS: left ventricular fractional shortening, IVRT: isovolumentric relaxation time, HR: heart rate. Values are mean/SD. N: is the number of rats at each time point. P-values: all significant values are shown (n.s. no significant; p>0.1). Colored values (blue in CDC-EV and red in PBS groups, respectively) represent the significance of the difference between baseline and the specific time-point within the group estimated with paired Student’s t-test. | | | | | | | | |

## Slide 4
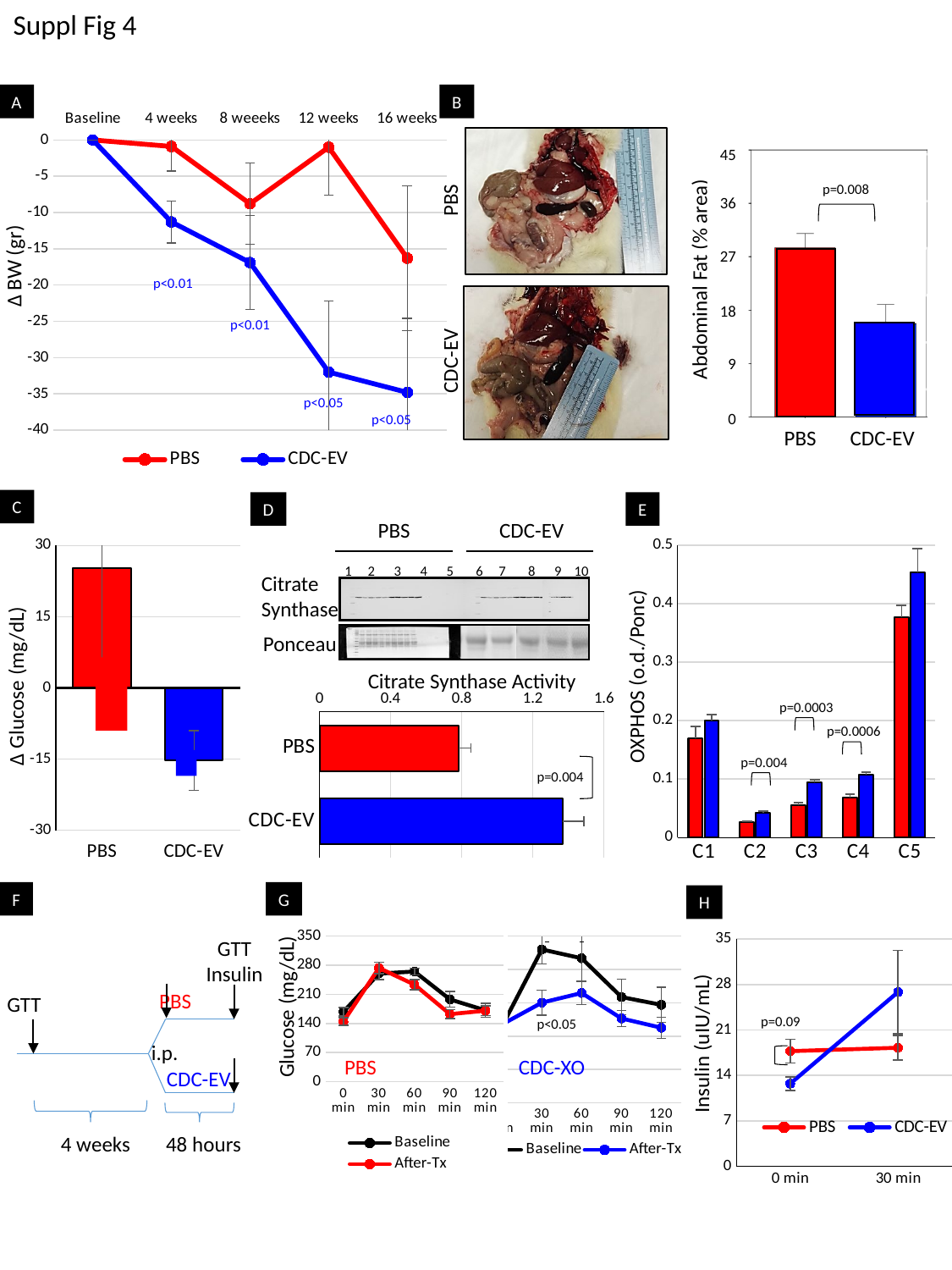

Suppl Fig 4
A
B
### Chart
| Category | PBS | CDC-EV |
|---|---|---|
| Baseline | 0.0 | 0.0 |
| 4 weeks | -0.9 | -11.3 |
| 8 weeeks | -8.8 | -16.9 |
| 12 weeks | -1.0 | -32.0 |
| 16 weeks | -16.3 | -34.8 |45
36
27
18
9
0
p=0.008
PBS CDC-EV
Abdominal Fat (% area)
Δ BW (gr)
p<0.01
 CDC-EV PBS
p<0.01
p<0.05
p<0.05
C
D
E
CDC-EV
PBS
 1 2 3 4 5 6 7 8 9 10
Citrate
Synthase
Ponceau
### Chart
| Category | PBS | Neo |
|---|---|---|
| C1 | 0.17 | 0.2 |
| C2 | 0.026 | 0.042 |
| C3 | 0.056 | 0.094 |
| C4 | 0.069 | 0.107 |
| C5 | 0.377 | 0.454 |
### Chart
| Category | |
|---|---|
| PBS | 25.2 |
| CDC-EV | -15.3 |
Δ Glucose (mg/dL)
OXPHOS (o.d./Ponc)
Citrate Synthase Activity
### Chart
| Category | |
|---|---|
| PBS | 0.782 |
| CDC-EV | 1.368 |p=0.0003
p=0.0006
p=0.004
p=0.004
G
F
H
### Chart
| Category | Baseline | After-Tx |
|---|---|---|
| 0 min | 168.16666666666666 | 144.66666666666666 |
| 30 min | 260.0 | 273.5 |
| 60 min | 265.0 | 233.5 |
| 90 min | 198.0 | 162.5 |
| 120 min | 171.6 | 170.83333333333334 |
### Chart
| Category | Baseline | After-Tx |
|---|---|---|
| 0 min | 160.6 | 164.4 |
| 30 min | 322.0 | 210.2 |
| 60 min | 303.4 | 230.5 |
| 90 min | 222.0 | 177.0 |
| 120 min | 205.6 | 157.5 |
### Chart
| Category | PBS | CDC-EV |
|---|---|---|
| 0 min | 17.74 | 12.725000000000001 |
| 30 min | 18.259999999999998 | 26.84 |GTT
Insulin
PBS
GTT
i.p.
CDC-EV
48 hours
4 weeks
Glucose (mg/dL)
p=0.09
p<0.05
Insulin (uIU/mL)
PBS
CDC-XO

## Slide 5
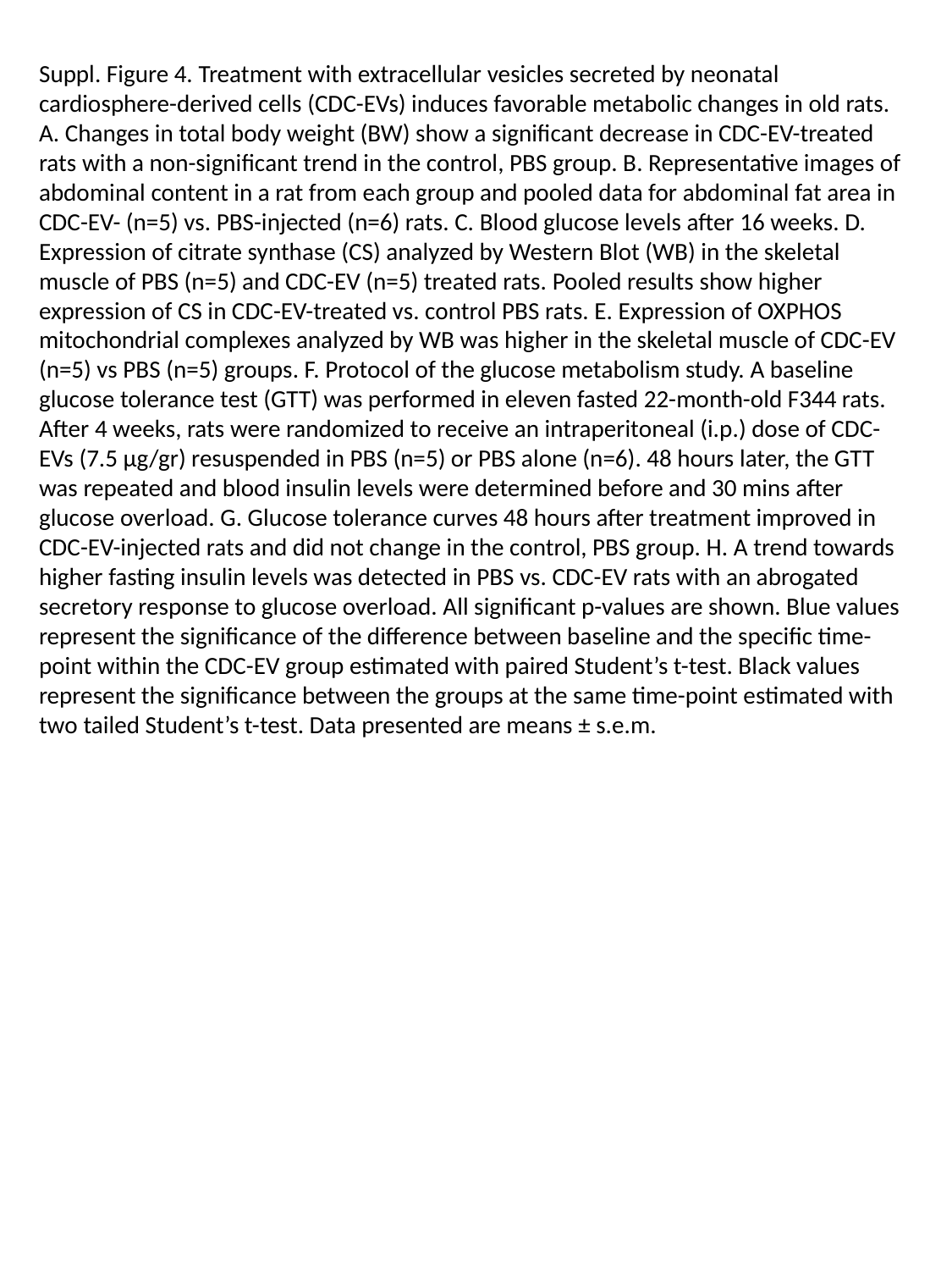

Suppl. Figure 4. Treatment with extracellular vesicles secreted by neonatal cardiosphere-derived cells (CDC-EVs) induces favorable metabolic changes in old rats. A. Changes in total body weight (BW) show a significant decrease in CDC-EV-treated rats with a non-significant trend in the control, PBS group. B. Representative images of abdominal content in a rat from each group and pooled data for abdominal fat area in CDC-EV- (n=5) vs. PBS-injected (n=6) rats. C. Blood glucose levels after 16 weeks. D. Expression of citrate synthase (CS) analyzed by Western Blot (WB) in the skeletal muscle of PBS (n=5) and CDC-EV (n=5) treated rats. Pooled results show higher expression of CS in CDC-EV-treated vs. control PBS rats. E. Expression of OXPHOS mitochondrial complexes analyzed by WB was higher in the skeletal muscle of CDC-EV (n=5) vs PBS (n=5) groups. F. Protocol of the glucose metabolism study. A baseline glucose tolerance test (GTT) was performed in eleven fasted 22-month-old F344 rats. After 4 weeks, rats were randomized to receive an intraperitoneal (i.p.) dose of CDC-EVs (7.5 μg/gr) resuspended in PBS (n=5) or PBS alone (n=6). 48 hours later, the GTT was repeated and blood insulin levels were determined before and 30 mins after glucose overload. G. Glucose tolerance curves 48 hours after treatment improved in CDC-EV-injected rats and did not change in the control, PBS group. H. A trend towards higher fasting insulin levels was detected in PBS vs. CDC-EV rats with an abrogated secretory response to glucose overload. All significant p-values are shown. Blue values represent the significance of the difference between baseline and the specific time-point within the CDC-EV group estimated with paired Student’s t-test. Black values represent the significance between the groups at the same time-point estimated with two tailed Student’s t-test. Data presented are means ± s.e.m.

## Slide 6
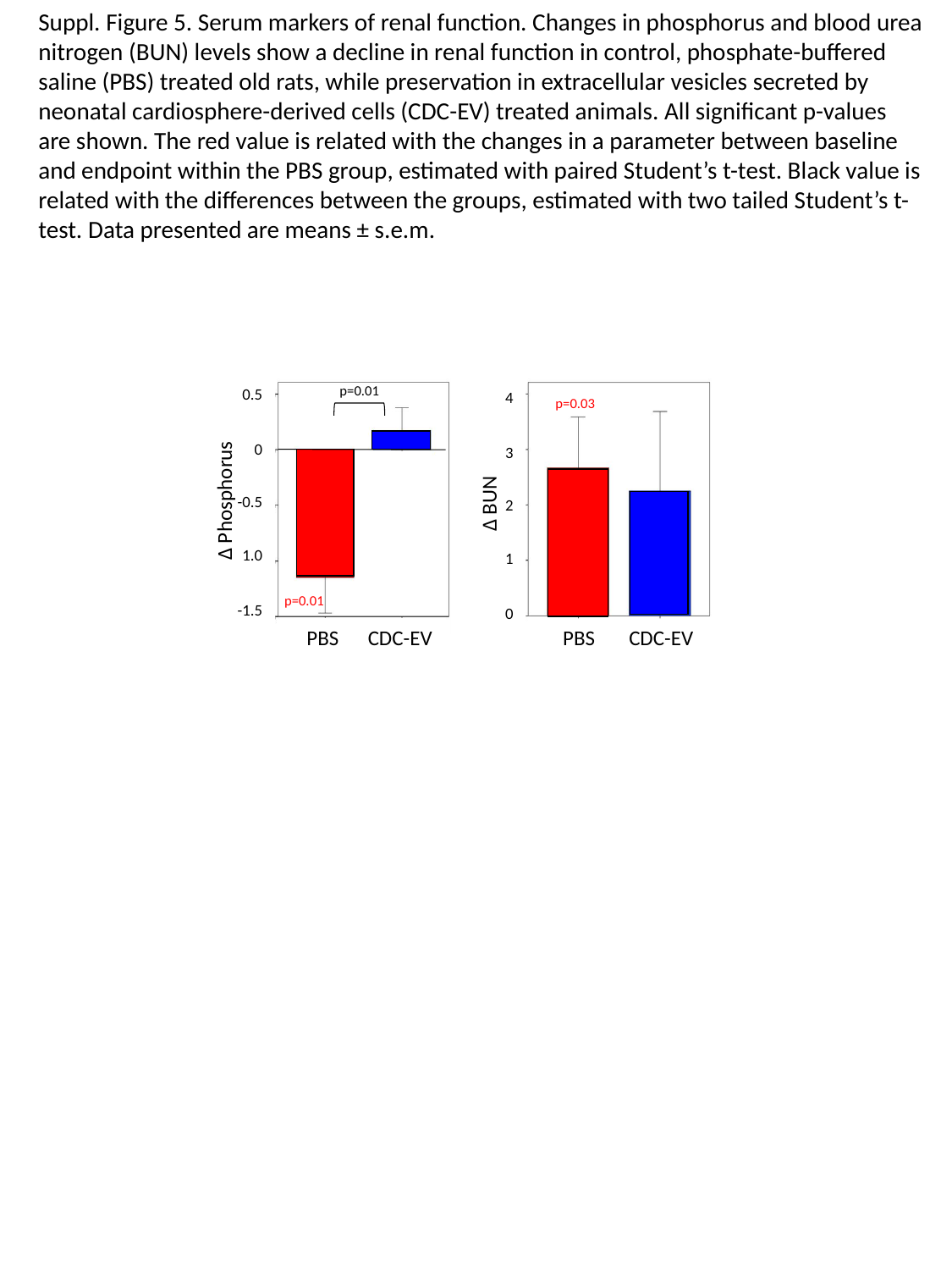

Suppl. Figure 5. Serum markers of renal function. Changes in phosphorus and blood urea nitrogen (BUN) levels show a decline in renal function in control, phosphate-buffered saline (PBS) treated old rats, while preservation in extracellular vesicles secreted by neonatal cardiosphere-derived cells (CDC-EV) treated animals. All significant p-values are shown. The red value is related with the changes in a parameter between baseline and endpoint within the PBS group, estimated with paired Student’s t-test. Black value is related with the differences between the groups, estimated with two tailed Student’s t-test. Data presented are means ± s.e.m.
p=0.01
0.5
0
-0.5
1.0
-1.5
p=0.01
PBS CDC-EV
Δ Phosphorus
4
3
2
1
0
p=0.03
Δ BUN
PBS CDC-EV

## Slide 7
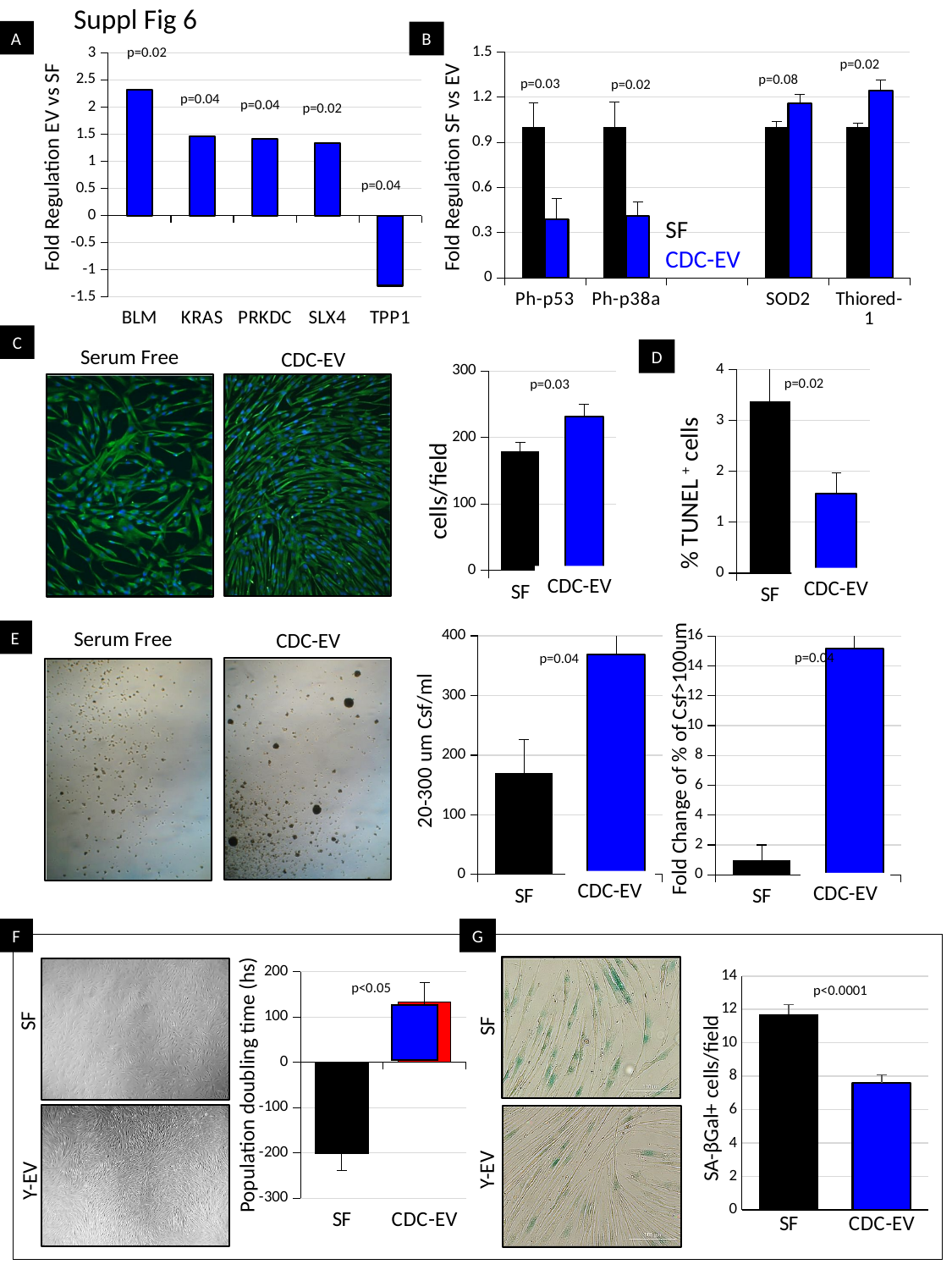

Suppl Fig 6
A
B
p=0.02
### Chart
| Category | SF | XO |
|---|---|---|
| Ph-p53 | 0.99999996925 | 0.38847852374999997 |
| Ph-p38a | 1.000037288 | 0.4114895295 |
| | None | None |
| SOD2 | 1.0000000655 | 1.15772101675 |
| Thiored-1 | 1.0000000835 | 1.2454190697499998 |
### Chart
| Category | |
|---|---|
| BLM | 2.3124 |
| KRAS | 1.4551 |
| PRKDC | 1.4186 |
| SLX4 | 1.3294 |
| TPP1 | -1.2933 |p=0.02
p=0.08
p=0.03
p=0.02
p=0.04
p=0.04
p=0.02
Fold Regulation SF vs EV
Fold Regulation EV vs SF
p=0.04
SF
CDC-EV
C
Serum Free
CDC-EV
D
### Chart
| Category | % Tunel+cell |
|---|---|
| SF | 3.3797993521996146 |
| XO | 1.566325653341069 |p=0.02
% TUNEL + cells
CDC-EV
### Chart
| Category | Cell N |
|---|---|
| SF | 179.11111111111111 |
| XO | 230.84615384615384 |p=0.03
cells/field
CDC-EV
Serum Free
E
CDC-EV
### Chart
| Category | Csf/ml |
|---|---|
| SF | 170.43939393333332 |
| XO | 368.1333333333334 |
### Chart
| Category | Fold >100 |
|---|---|
| SF | 1.0 |
| XO | 15.178571428571427 |p=0.04
p=0.04
20-300 um Csf/ml
Fold Change of % of Csf>100um
CDC-EV
CDC-EV
F
G
### Chart
| Category | |
|---|---|
| SF | -202.03 |
| CDC-EV | 132.5 |
### Chart
| Category | |
|---|---|
| SF | 11.7 |
| CDC-EV | 7.6 |p<0.05
p<0.0001
SF
SF
Population doubling time (hs)
SA-βGal+ cells/field
Y-EV
Y-EV

## Slide 8
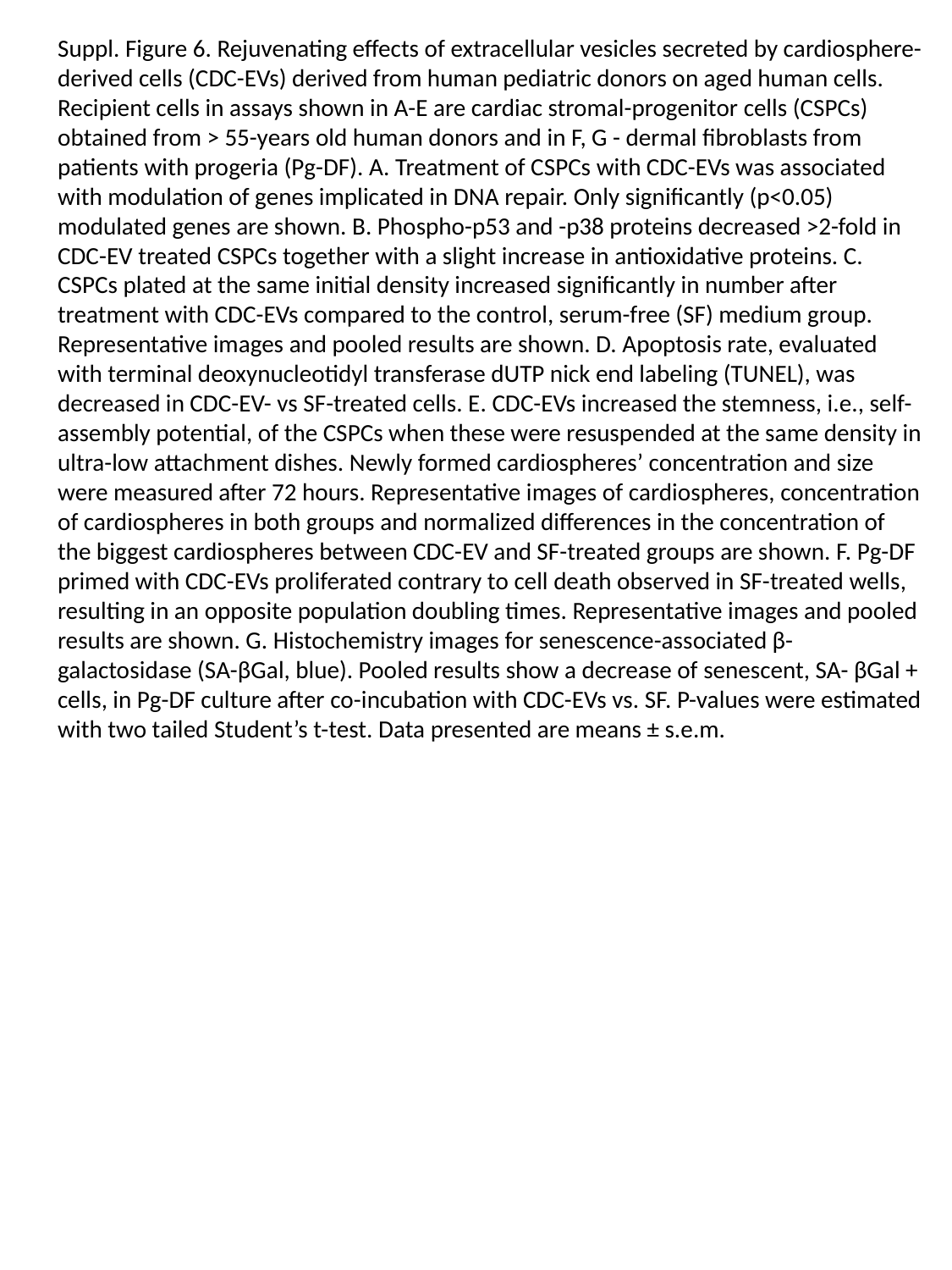

Suppl. Figure 6. Rejuvenating effects of extracellular vesicles secreted by cardiosphere-derived cells (CDC-EVs) derived from human pediatric donors on aged human cells. Recipient cells in assays shown in A-E are cardiac stromal-progenitor cells (CSPCs) obtained from > 55-years old human donors and in F, G - dermal fibroblasts from patients with progeria (Pg-DF). A. Treatment of CSPCs with CDC-EVs was associated with modulation of genes implicated in DNA repair. Only significantly (p<0.05) modulated genes are shown. B. Phospho-p53 and -p38 proteins decreased >2-fold in CDC-EV treated CSPCs together with a slight increase in antioxidative proteins. C. CSPCs plated at the same initial density increased significantly in number after treatment with CDC-EVs compared to the control, serum-free (SF) medium group. Representative images and pooled results are shown. D. Apoptosis rate, evaluated with terminal deoxynucleotidyl transferase dUTP nick end labeling (TUNEL), was decreased in CDC-EV- vs SF-treated cells. E. CDC-EVs increased the stemness, i.e., self-assembly potential, of the CSPCs when these were resuspended at the same density in ultra-low attachment dishes. Newly formed cardiospheres’ concentration and size were measured after 72 hours. Representative images of cardiospheres, concentration of cardiospheres in both groups and normalized differences in the concentration of the biggest cardiospheres between CDC-EV and SF-treated groups are shown. F. Pg-DF primed with CDC-EVs proliferated contrary to cell death observed in SF-treated wells, resulting in an opposite population doubling times. Representative images and pooled results are shown. G. Histochemistry images for senescence-associated β-galactosidase (SA-βGal, blue). Pooled results show a decrease of senescent, SA- βGal + cells, in Pg-DF culture after co-incubation with CDC-EVs vs. SF. P-values were estimated with two tailed Student’s t-test. Data presented are means ± s.e.m.

## Slide 9
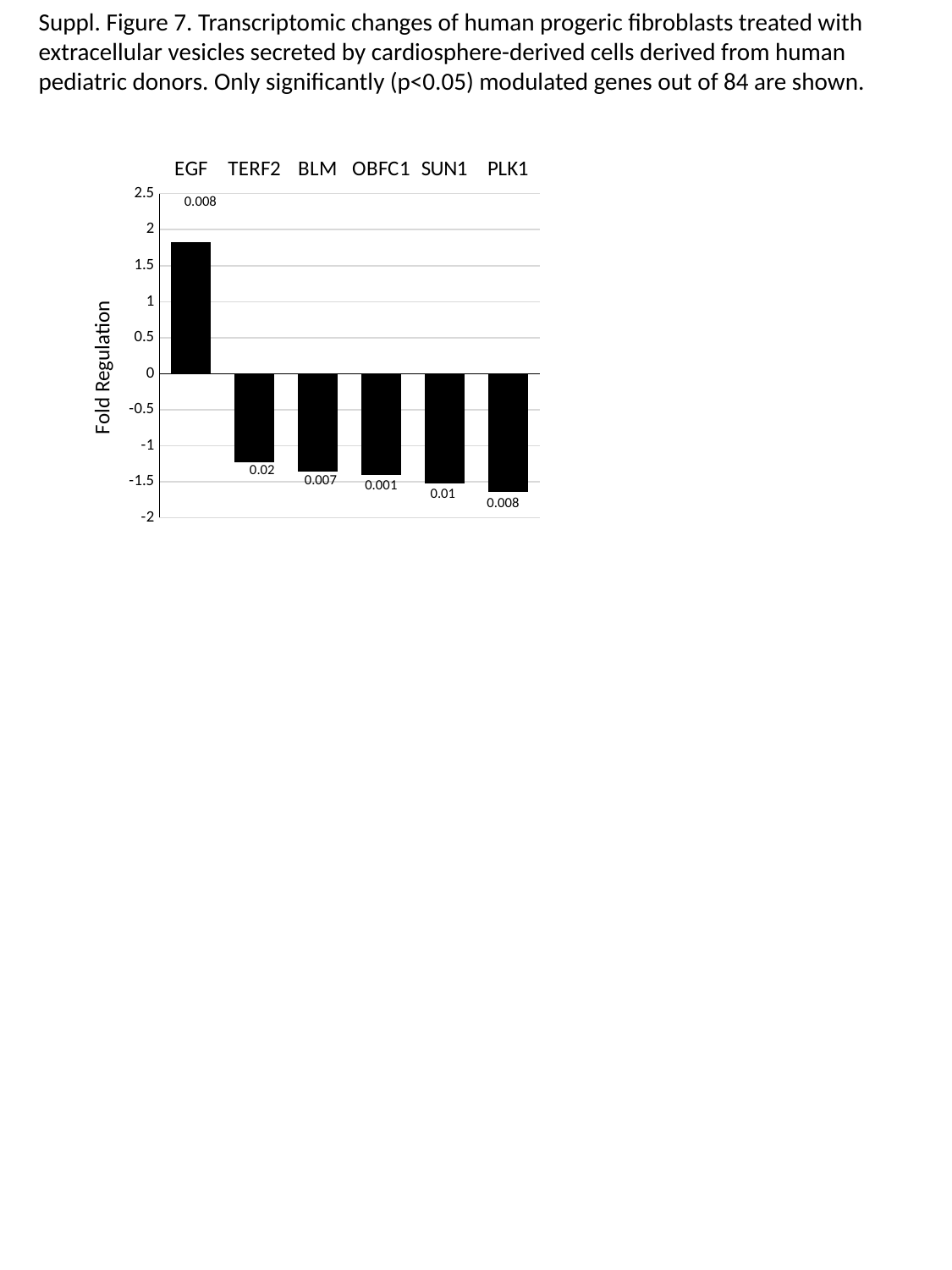

Suppl. Figure 7. Transcriptomic changes of human progeric fibroblasts treated with extracellular vesicles secreted by cardiosphere-derived cells derived from human pediatric donors. Only significantly (p<0.05) modulated genes out of 84 are shown.
### Chart
| Category | |
|---|---|
| EGF | 1.8207 |
| TERF2 | -1.2301 |
| BLM | -1.358 |
| OBFC1 | -1.404 |
| SUN1 | -1.5231 |
| PLK1 | -1.647 |0.008
Fold Regulation
0.02
0.007
0.001
0.01
0.008

## Slide 10
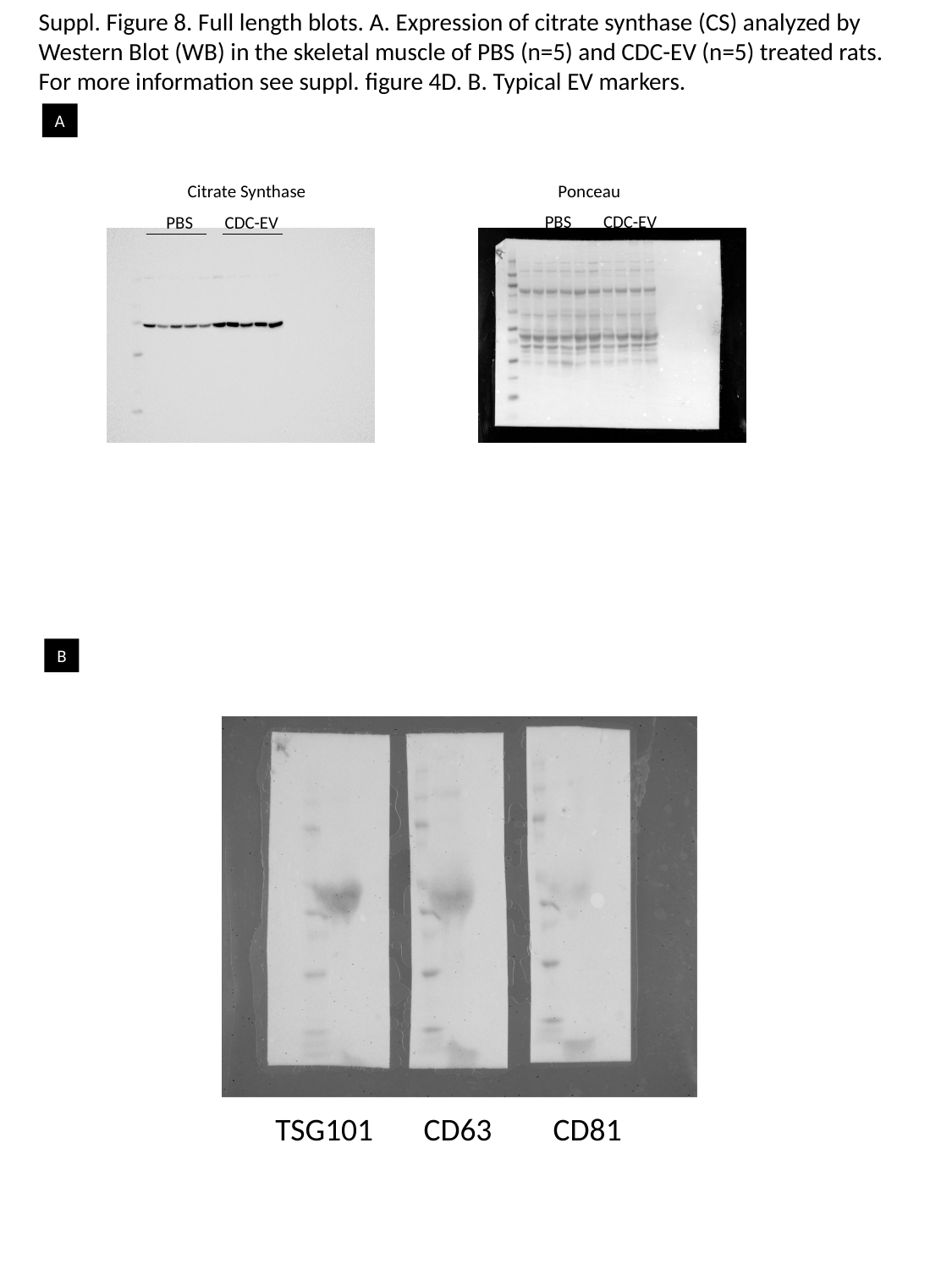

Suppl. Figure 8. Full length blots. A. Expression of citrate synthase (CS) analyzed by Western Blot (WB) in the skeletal muscle of PBS (n=5) and CDC-EV (n=5) treated rats. For more information see suppl. figure 4D. B. Typical EV markers.
A
Citrate Synthase Ponceau
PBS CDC-EV
PBS CDC-EV
B
TSG101
CD63
CD81

## Slide 11
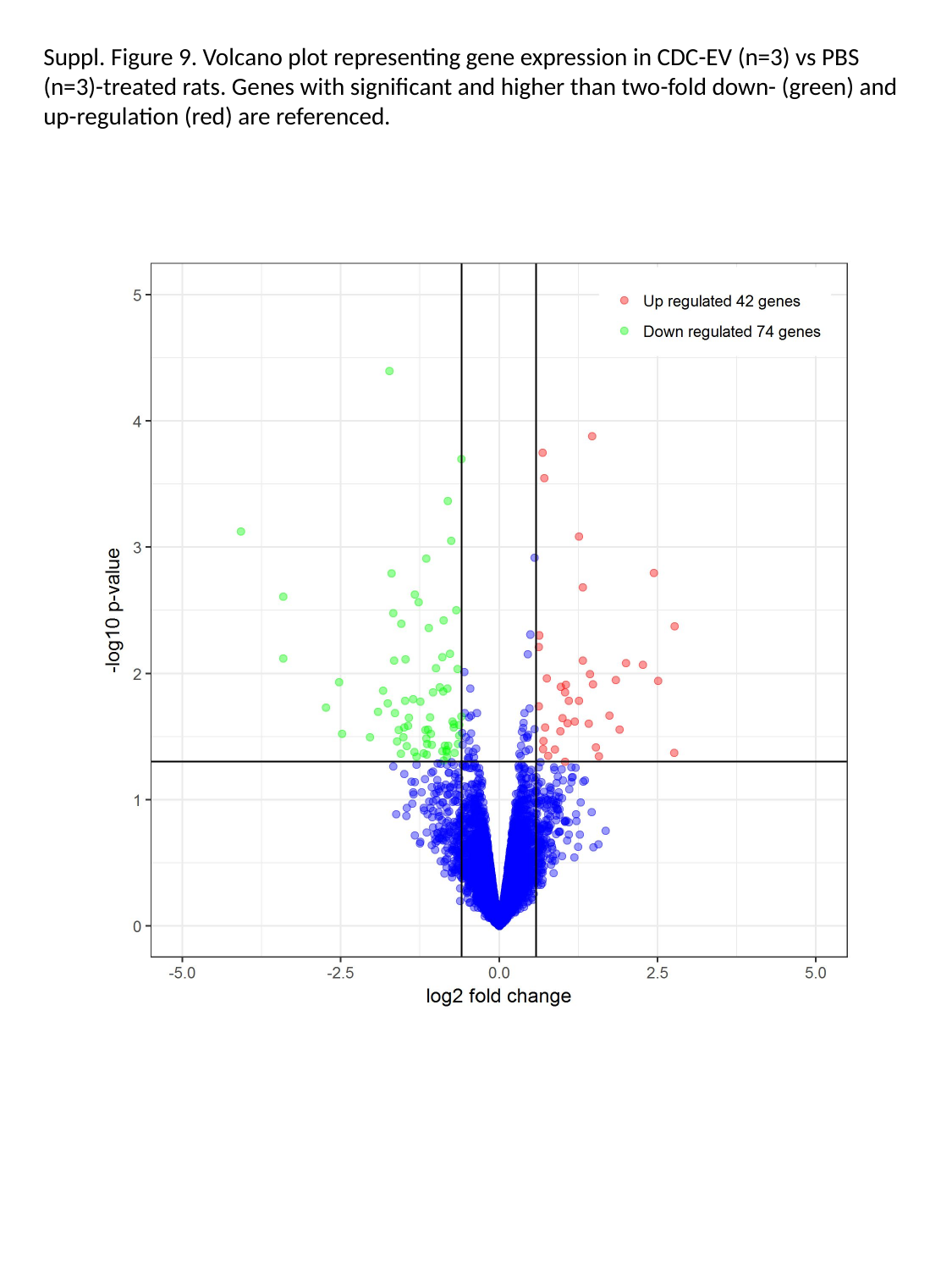

Suppl. Figure 9. Volcano plot representing gene expression in CDC-EV (n=3) vs PBS (n=3)-treated rats. Genes with significant and higher than two-fold down- (green) and up-regulation (red) are referenced.
